# The RNA m^6^A reader IGF2BP3 regulates NFAT1/IRF1 axis-mediated anti-tumor activity in gastric cancer

**DOI:** 10.1038/s41419-024-06566-0

**Published:** 2024-03-06

**Authors:** Lichen Ge, Yalan Rui, Cheng Wang, Yingmin Wu, Hongsheng Wang, Junjun Wang

**Affiliations:** 1 Department of Clinical Laboratory, Jinling Hospital, Medical School of Nanjing University, Nanjing, 210002 China; 2 Department of Laboratory Medicine, Third Affiliated Hospital of Sun Yat-sen University, Guangzhou, 510630 China; 3 Key Laboratory of Chiral Molecule and Drug Discovery, School of Pharmaceutical Sciences, Sun Yat-sen University, Guangzhou, 510006 China; 4 Guizhou Provincial Key Laboratory of Pathogenesis & Drug Research on Common Chronic Diseases, Department of Physiology, School of Basic Medical Sciences, Guizhou Medical University, Guiyang, 550009 China

**Keywords:** Gastric cancer, Oncogenesis

## Abstract

*N*^6^-methyladenosine (m^6^A) and its associated reader protein insulin like growth factor 2 mRNA binding protein 3 (IGF2BP3) are involved in tumor initiation and progression via regulating RNA metabolism. This study aims to investigate the biological function and clinical significance of IGF2BP3 in gastric cancer (GC). The clinical significance of IGF2BP3 was evaluated using tumor related databases and clinical tissues. The biological role and molecular mechanism of IGF2BP3 in GC progression were investigated by multi-omics analysis including Ribosome sequence (Ribo-seq), RNA sequence (RNA-seq) and m^6^A sequence (m^6^A-seq) combined with gain- and loss- of function experiments. IGF2BP3 expression is significantly elevated in GC tissues and associated with poor prognosis of GC patients. Knockdown of IGF2BP3 significantly weakens the migration and clonogenic ability, promotes the apoptosis, inhibits translation, and suppresses in vitro growth and progression of GC cells. Mechanistically, IGF2BP3 regulates the mRNA stability and translation of the nuclear factor of activated T cells 1(NFAT1) in a m^6^A dependent manner. Then NFAT1 induced by IGF2BP3 acts as a transcription factor (TF) to negatively regulates the promoter activities of interferon regulatory factor 1 (IRF1) to inhibit its expression. Inhibition of IGF2BP3-induced expression of IRF1 activates interferon (IFN) signaling pathway and then exerts its anti-tumor effect. Elevated IGF2BP3 promotes in vivo and in vitro GC progression via regulation of NFAT1/IRF1 pathways. Targeted inhibition of IGF2BP3 might be a potential therapeutic approach for GC treatment.

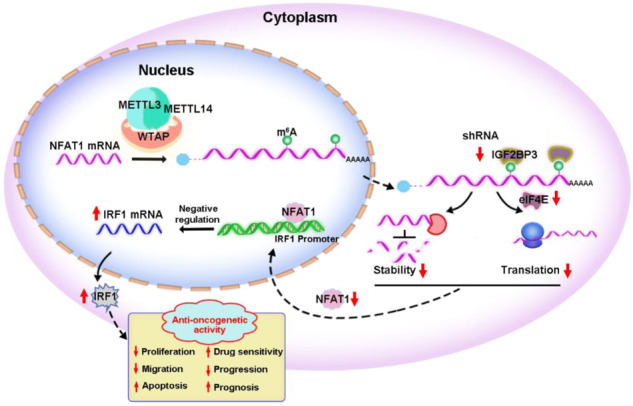

## Introduction

Gastric cancer (GC) is the fifth most common cancer and the fourth leading cause among cancer related deaths worldwide [1]. In China, data submitted from high-quality cancer registries estimated that approximately 403,000 people were newly diagnosed with GC and almost 291,000 died nationwide in 2015 [2]. Thus, there is an urgent need to develop novel and effective therapeutic targets for GC.

*N*^6^-methyladenosine (m^6^A) is the most abundant chemical modification in eukaryotic mRNAs [3–5]. The m^6^A methylation is a dynamic and reversible process regulated by methyltransferases (called writers: METTL3, METTL14 and WTAP and so on) and demethylases (called erasers: FTO and ALKBH5 and so on) [6–9]. The m^6^A-binding proteins (called reader: YTHDF1/2/3, YTHDC1/2, IGF2BP1/2/3 and so on) could bind to the m^6^A site to affect the fate of mRNA by regulating its nuclear transport, alternative splicing, degradation and translation [10]. Dysregulation of m^6^A methylation has been proved to be associated with various human carcinogenesis [11]. Aberrant expressions of m^6^A methyltransferases and demethylases were reported to promote development and progression of GC [12, 13]. However, the biological significances of m^6^A reader proteins and theirs underlying molecular mechanisms in GC are largely unclear.

Interferon (IFNs) reflect powerful antitumor actions [14]. Interferon regulatory factors (IRFs) are key transcription factors (TFs) of IFN signaling regulation network. As a well-known tumor suppressor, IRF1 exerts multiple biological functions in cancer cells, including antiproliferation, initiation of apoptosis and cell cycle arrest [15]. *IRF1* locus showed high frequency heterozygous loss in GC patients [16]. Functionally inactivating point mutation in the *IRF-1* locus were observed in human GC [17]. However, the cross talk between m^6^A methylation and IRF1 has not been elucidated.

Nuclear factor of activated T cells 1 (NFAT1) is the major NFAT isoform, which plays the vital roles in the immune system and inflammatory response [18, 19]. Accumulating evidences showed NFAT1 is highly expressed in various human cancers, and contributes to tumorigenesis and progression by regulating cancer cell proliferation, cell cycle, apoptosis, migration and invasion [20–23].

In this study, we comprehensively investigated the effects of m^6^A reader protein IGF2BP3 on the malignant biological behavior of GC and elucidated its molecular mechanism and propose that IGF2BP3 may be a novel therapeutic target for GC progression.

## Materials and methods

### Tissue samples collection

GC and adjacent non-tumor tissue samples were collected from 5 patients who underwent gastrectomy at the general surgery of Jinling Hospital of Nanjing University. The fresh tissues were immediately placed in RNAlater RNA Stabilization Reagent to stabilize RNA, then stored at −20 °C until RNA isolation. The fresh tissues were immediately stored at liquid nitrogen until protein isolation. The newly diagnosed GC patients didn’t receive radiochemotherapy before surgery. Informed consent was obtained for all individuals. Ethics approval was obtained from the Ethics Committee of Jinling Hospital of Nanjing University.

### Database analysis

The Oncomine [24] (https://www.oncomine.org), GEPIA [25] (http://gepia.cancer-pku.cn) and OncoDB [26] (https://oncodb.org) databases were used to examine gene expression in tumor and adjacent non-tumor tissues. The Kaplan-Meier Plotter [27] (http://www.kmplot.com/analysis/) and UALCAN [28] (https://ualcan.path.uab.edu/) databases were used to validate the correlation between expression of genes and clinical characteristics of GC patients.

### Cell culture

Human GC cell lines (MKN-45, AGS) were obtained from Kunming Cell Bank (Chinese Academy of Sciences) and cultured as per the established protocol [29]. All cell lines were authenticated using short tandem repeat (STR) profiling analysis (Shanghai Biowing Applied Biotechnology Co. Ltd) and were negative for mycoplasma as measured by Myco-Blue Mycoplasma Detector (Vazyme) before experiment. We established IGF2BP3 and METTL3 knockdown GC cells using the short hairpin RNA (shRNA) lentiviral vector and generated stable IGF2BP3 and METTL3 knockdown MKN-45 and AGS cells respectively by puromycin selection.

### Plasmid, siRNA, shRNA and generation of stable cell lines

The NFAT1 over-expression plasmid was kindly provided by Prof. Na Liu at the Sun Yat-sen University Cancer Center. Psin empty plasmid was used as the vector control for analysis. Cells were transfected by using Lipofectamine 3000 reagent (Thermo). IGF2BP3, IRF1 and NFAT1 siRNA were used to induce gene silencing. A nontargeting siRNA was used as a negative control. Cells were transfected by using Lipofectamine RNAiMAX reagent (Thermo). Two shRNAs targeting IGF2BP3 (shIGF2BP3-1, shIGF2BP3-2), one shRNA targeting METTL3 (shMETTL3) and a nontargeting shRNA (shNC) serving as a negative control were purchased from Obio Technology. The stable IGF2BP3 and METTL3 knockdown GC cells were generated by puromycin selection. The expressions of IGF2BP3 and METTL3 were checked by western blot analysis. The nucleotide sequence of siRNA and shRNA used in this study are listed in Table S1.

### Wound-healing assay

Cells were seeded and cultured until a 90% confluent monolayer was formed. Cells were then scratched by a sterile pipette tip and treated as indicated in the text in the FBS-free medium. Cell migration distances into the scratched area were measured in 10 randomly chosen fields under a microscope.

### Transwell assay

Transwell chambers (polycarbonate filters with pore size of 8 µm, Corning) were used to evaluate the migration capacity of GC cells. Cells (1.0 × 10^5^ per well) suspended in 200 μL culture medium contained 1% FBS were placed in the upper compartment, and 600 μL of culture medium supplemented with 10% FBS were added to the lower chamber. The cells in the transwell chambers were fixed and stained with 0.25% crystal violet after 24 h of incubation. The number of migrated cells to the lower chamber were counted.

### Colony formation assay

Cells (500 per well) were seeded in the 6-well plate, and the medium was replaced every 3 days. Colonies were stained with 0.25% crystal violet after two weeks.

### EdU staining proliferation assay

Cells (4.0 × 10^5^ per well) were seeded in the 6-well plate, cells proliferation status were performed using the BeyoClick™ EdU Cell Proliferation Kit with Alexa Fluor 555 (Beyotime) in accordance with the manufacturer’s instruction.

### Apoptosis assay

Cells were stained with 50 μg/mL Annexin V-FITC (Becton, Dickinson and Company) and 10 μg/mL PI (Becton, Dickinson and Company) for 10 min at 37 °C, then analyzed for apoptosis by using guava easyCyte (Millipore).

### Subcellular fractionation

To determine the cellular localization of protein and mRNA, cytoplasmic and nuclear proteins and RNAs were isolated using PARIS Kit (Thermo) according to the manufacturer’s instructions. The extracted proteins and RNAs were subjected to western blot and RT-qPCR analysis, respectively. The GAPDH and Histone H3 were used as cytoplasmic control and nuclear control respectively for western blot analysis. The GAPDH mRNA was used as cytoplasmic control and U6 RNA as nuclear control for RT-qPCR analysis.

### Chromatin immunoprecipitation (ChIP) qPCR

The ChIP assay was performed using the Agarose ChIP Kit (Thermo) following the manufacturer’s instructions. Anti-NFAT1 or immunoglobulin G (IgG) antibody were added to the supernatant and incubated overnight at 4 °C. The DNA fragments were released from the bound chromatin after crosslinking and micrococcal nuclease digestion, immunoprecipitated, and finally eluted in 50 μL of DNA column elution solution. The primers used for the qPCR in this study are listed in Table S2.

### RNA-binding protein immunoprecipitation (RIP)-RT-qPCR

The RIP experiment was performed using the Magna RIP^™^ Quad RNA-Binding Protein Immunoprecipitation Kit (Millipore) according to the manufacturer’s instructions. Briefly, cells (1.0 × 10^7^) were irradiated twice with 400 mJ/cm^2^ at 254 nm by Stratalinker on ice and lysed with 200 μL of RIP lysis buffer, of which 5 μL of supernatant was collected as input, and 150 μL of supernatant was incubated with IGF2BP3 or eIF4E antibody, or IgG-conjugated protein A/G magnetic beads in 500 μL IP buffer supplemented with RNase inhibitors at 4 °C overnight. Bound RNAs were immunoprecipitated with beads and treated with 100 µl of elution buffer at 50 °C for 2 h, and RNA was purified, then further analyzed by RT-qPCR analysis. IP enrichment ratio of specific transcript was calculated as ratio of its amount in IP to that in the input, yielded from same amounts of cells.

### RNA pulldown assay

The IRF1 and MYC expression plasmids containing T7 promoter were used to generate linearized DNA template. Biotin-labeled IRF1 mRNA and MYC mRNA were transcribed with Biotin RNA Labeling Mix and T7 RNA polymerase (RiboBio). After purification, biotinylated RNAs (3 ug) were incubated with cell lysates at 4 °C overnight. MyOne™ Streptavidin C1 Beads (Thermo) were added to each tube, and incubated for 1 h at room temperature. The enriched proteins were subjected to western blot analysis.

### RNA stability assay

To measure RNA stability in shNC and shIGF2BP3 MKN-45 cells, actinomycin D (ACTD, 5 μg/ml) was added to serum-free culture medium. After incubation at the indicated times, cells were collected, and RNA was isolated for RT-qPCR analysis. The mRNA expressions of IRF1 and NFAT1 were checked, GAPDH was used for normalization.

### Luciferase reporter assay

Promoter activities of IRF1 in cells were measured by luciferase assay. Briefly, cells were transfected with pGL3-IRF1-WT-Luc or pGL3-IRF1-Mut1-Luc or pGL3-IRF1-Mut2-Luc containing the −820 ~ +138 sequence of the IRF1 promoter. Transfection efficiency was normalized by co-transfection with pRL-TK. After incubation at the indicated times, luciferase activities were measured using the Dual Luciferase Reporter Gene Assay Kit (Beyotime) according to the manufacturer’s instructions. Renilla luciferase (R-luc) was used to normalize firefly luciferase (F-luc) activity to evaluate the reporter transcription.

To evaluate the potential roles of m^6^A in 3’UTR for NFAT1 expression, the wild type or mutant-1/-2/-3 of 3’UTR of NFAT1 was inserted behind the F-luc coding region of pmirGLO plasmid to generate pmirGLO-NFAT1-3’UTR-WT, pmirGLO-NFAT1-3’UTR-Mut1, pmirGLO-NFAT1-3’UTR-Mut2, and pmirGLO-NFAT1-3’UTR-Mut3, respectively. Both the plasmids were transfected into shNC and shIGF2BP3 cells for the indicated times, the F-luc and R-luc were assayed by Dual Luciferase Reporter Gene Assay Kit (Beyotime). R-luc was used to normalize F-luc activity to evaluate the potential roles of m^6^A in 3’UTR for NFAT1 expression.

### RNA extraction and quantitative real-time PCR

Total RNA was isolated from tissue samples using the miRNeasy Micro Kit (QIAGEN) according to the manufacturer’s recommendation. Total cellular RNA was isolated using TRIzol reagent (Thermo). The yield and purity of RNA were measured by NanoDrop 2000 (Thermo). cDNA was generated using the PrimeScript™ RT Master Mix (Takara). Quantitative real-time PCR using TB Green™ Premix Ex Taq™ II (Takara) was performed on a CFX96 Touch™ Real-Time PCR Detection System (Bio-Rad). The cycle parameters were as follows: 95 °C for 5 min followed by 40 cycles of 95 °C for 10 s, 60 °C for 30 s, and 65 °C for 5 s. The expressions of targeted genes were normalized to 18 S for tissues and GAPDH for cells respectively. The relative expression levels were calculated by the 2^−ΔCt^ method. Primers of targeted genes are listed in Table S2.

### RNA m^6^A quantification

The m^6^A levels in total RNA were measured by use of EpiQuik m^6^A RNA Methylation Quantification Kit (Colorimetric) (Epigentek) according to the manufacturer’s instructions and previously published protocol [29].

### Polysome profiling

The fraction of ribosome was separated by centrifugation in a sucrose gradient. Cells were treated with Cycloheximide (CHX, 100 μg/mL) for 10 min at 37 °C, then washed with precooling PBS contained CHX (100 μg/mL) and lysed on ice in lysis buffer. The lysate was collected, centrifuged at 15,000 g for 15 min, the supernatant was separated by 5/50% (w/v) sucrose gradient solution at 4 °C for 2 h at 160,000 g (Beckman, rotor SW28). The sample was then fractioned and analyzed by Gradient Station (BioCamp) equipped with ECONO UV monitor (BioRad) and fraction collector (FC203B, Gilson). RNA was purified by Trizol reagent from each fraction and subjected to qRT-PCR analysis.

### Ribo-seq

Preparation of ribosome footprints for Ribo-seq experiments was performed according to previous report with minor modifications [30]. MKN-45 cells were grown to 90% confluency in 15 cm diameter culture dishes prepared for cell lysis. The culture media was removed, and cells were washed twice with 5 mL ice cold PBS containing 100 μg/mL CHX. After removing PBS, 400 µL of ice cold lysis buffer (20 mM Tris-HCl at pH 7.4, 150 mM NaCl, 5 mM MgCl_2_, 1 mM DTT, 1% Triton X-100) supplemented with 100 μg/mL CHX, 500 U/mL RNase inhibitor (Thermo) and protease inhibitor Cocktail (Roche) (added freshly) was dripped onto the plates. Cells were scraped then incubated on ice in lysis buffer for 15 min with pipetting to disperse the cells. The lysate then cleared by centrifugation at 15000 g for 10 min. The prepared supernatant was digested with RNase I (Thermo) for 45 min at room temperature. SUPERase·In RNase Inhibitor (Thermo) was then added to stop the reaction. RFPs (ribosome footprints) were purified using MicroSpin S-400 HR columns (GE Healthcare), then isolated using TRIzol reagent. To enrich the RFPs, Ribosomal RNAs were depleted from ribosome protected fragments (RPFs) using the Ribo-Zero mammalian kit (Illumina) following the manufacturer’s protocol then separated with a 17% 7.5 M urea-PAGE gel. RNA with sizes ranging from 26 nt to 30 nt were isolated and purified by PAGE. cDNA sequencing libraries were prepared using the TruSeq Ribo profile kit (Illumina) following the manufacturer’s instructions. Libraries were sequenced on the HiSeq X Ten platform (Illumina). Two replicates were analyzed for the shNC and shIGF2BP3 MKN-45 cells.

### RNA-seq and bioinformatics

Total RNAs from shNC and shIGF2BP3 MKN-45 cells were extracted using TRIzol reagent. After passing quality control, the libraries were generated and sequenced with the Illumina HiSeq 4000 (PE150) according to the protocol from the company (GENE DENOVO). RNA-seq reads were preceded by removing adapters using cutadapt, mapped to reference human genome sequence (Hg19). The read counts were expressed as FPKM. The differential expression between conditions was statistically assessed by R/Bioconductor package edgeR (version 3.0.8). Genes with *p* of < 0.05 and > 200 bp were called as differentially expressed.

For Gene Set Enrichment Analysis (GSEA), standard procedure (http://www.broadinstitute.org/gsea/doc/ GSEAUserGuideFrame.html) as described by GSEA user guide was performed. The Molecular Signature Database version 4.0 was used to compute overlaps between gene sets in Molecular Signature Database and our gene set.

### m^6^A-seq and data analysis

Total polyadenylated RNA was isolated from MKN-45 cells by using FastTrack MAGMaxi mRNA isolation kit (Thermo). RNA fragmentation, m^6^A RIP, and library preparation were conducted according to manufacturer’s instructions and previously published protocol [31]. The library was prepared by use of NEBNext Ultra Directional RNA Library Prep Kit (New England BioLabs). Each experiment was conducted with two biological replicates. m^6^A-seq data were analyzed according to protocols described before [31]. Significant peaks with FDR < 0.05 were annotated to RefSeq database (Hg19). Sequence motifs were identified by using Homer. Gene expression was calculated by Cufflinks using sequencing reads from input samples. Cuffdiff was used to find DE genes.

### m^6^A-RIP qPCR

1 μg m^6^A (Synaptic Systems) or IgG antibody were incubated with Protein G Magnetic beads in 1 × reaction buffer at 4 °C for 3 h, followed by incubation with 200 μg extracted RNA at 4 °C for 3 h. Incubation of RNA-antibody-conjugated beads with 100 µl elution buffer for 30 min at room temperature was used to elute the bound RNAs. The eluted RNAs were recovered by ethanol precipitation, and RNA concentration was measured with Qubit RNA HS Assay Kit (Thermo). Then 5 ng of the total RNA and m^6^A IP RNA were further analyzed by RT-qPCR. IP enrichment ratio of a transcript was calculated as the ratio of its amount in IP to that in the input yielded from same amounts of cells.

### Western blot analysis

Western blot analysis was performed as previously described [32]. The antibodies used in present study were: IGF2BP3 (Abcam, ab177477, 1:1000), β-Tubulin (Proteintech, 10094-1-AP, 1:1000), METTL3 (Abcam, ab195352, 1:1000), GAPDH (Proteintech, 60004-1-Ig, 1:1000), PARP (CST, 9532, 1:1000), Caspase-3 (CST, 14220, 1:1000), Cytochrome c (CST, 4280, 1:1000), IRF1 (CST, 8478, 1:1000), IRF2 (Abcam, ab124744, 1:1000), Histone H3 (Abbkine, ABL1070, 1:1000), STAT1 (CST, 14994, 1:1000), Phospho-STAT1 (CST, 9167, 1:1000), SP1 (Abcam, ab124804, 1:1000), NFAT1 (Abcam, ab92490, 1:1000), eIF4E (Proteintech, 11149-1-AP, 1:1000). β-Tubulin, GAPDH and Histone H3 were used as the loading control for tissues and cells respectively.

### Protein stability

CHX (100 μg/mL) was added to culture medium of the shNC and shIGF2BP3 MKN-45 cells at predetermined intervals. The protein stability of IRF1 was measured through western blot analysis.

### Immunohistochemistry (IHC)

IHC assay was performed as described previously [33]. The antibodies used in this study were: METTL3 (Abcam, ab195352, 1:500), METTL14 (Sigma, HPA038002, 1:500), WTAP (Proteintech, 60188-1-Ig, 1:500), IGF2BP3 (Proteintech, 14642-1-AP, 1:500), IRF1 (CST, 8478, 1:100), NFAT1 (CST, 5861, 1:500).

### Animal study

BALB/c nude mice (four weeks old) were purchased from Sun Yat-sen University Animal Center (Guangzhou, China). All animal experiments were performed according to protocols approved by Zhongshan School of Medicine Policy on Care and Use of Laboratory Animals. To establish human gastric cancer xenograft model in nude mice, shIGF2BP3 or shNC MKN-45 cells (5 × 10^6^ cells in 200 μl PBS) were injected into right flanks of athymic nude mice (*n* = 5, male: female=3:2). Mice were euthanized 30 days after cell injection or if the longest dimension of the tumors reached 2.0 cm before 30 days. Immediately following euthanasia, tumors were removed and weighed for use in histology and further studies. The tumor volume was calculated using the formula V = 1/2 × larger diameter × (smaller diameter).

### Statistics

Data were reported as mean ± SD from at least three independent experiments. For statistical analysis, two-tailed unpaired Student’s *t*-test between two groups and by one-way or two-way ANOVA followed by Bonferroni test for multiple comparison were performed. All statistical tests were two-sided. Data analysis was carried out using GraphPad Prism. A *p*-value of < 0.05 was considered significant. *p < 0.05, **p < 0.01; NS, no significant.

## Results

### Elevated IGF2BP3 expression is associated with poor prognosis of GC patients

To identify the role of m^6^A modification in GC, we first examined the expressions of m^6^A related enzymes in GC tissues and normal gastric mucosa tissues. We found that the expressions of methyltransferases METTL3 and METTL14 were significantly upregulated in GC tissues compared to normal mucosa tissues respectively, based on Cho Gastric, Wang Gastric and DErrico Gastric datasets from the Oncomine database (Fig. S1). RT-qPCR and IHC assays showed that expressions of METTL3 were elevated in GC tumor tissues compared to para-tumor tissues (Figure S2A, B). The m^6^A levels in GC tumor tissues were also distinctly higher than those in para-tumor tissues (Fig. S2C). Using the Kaplan‐Meier Plotter, we found that GC patients with increased mRNA levels of METTL3, METTL14 and WTAP showed worse overall survival (OS) (Fig. S2D).

**Fig. 1:**
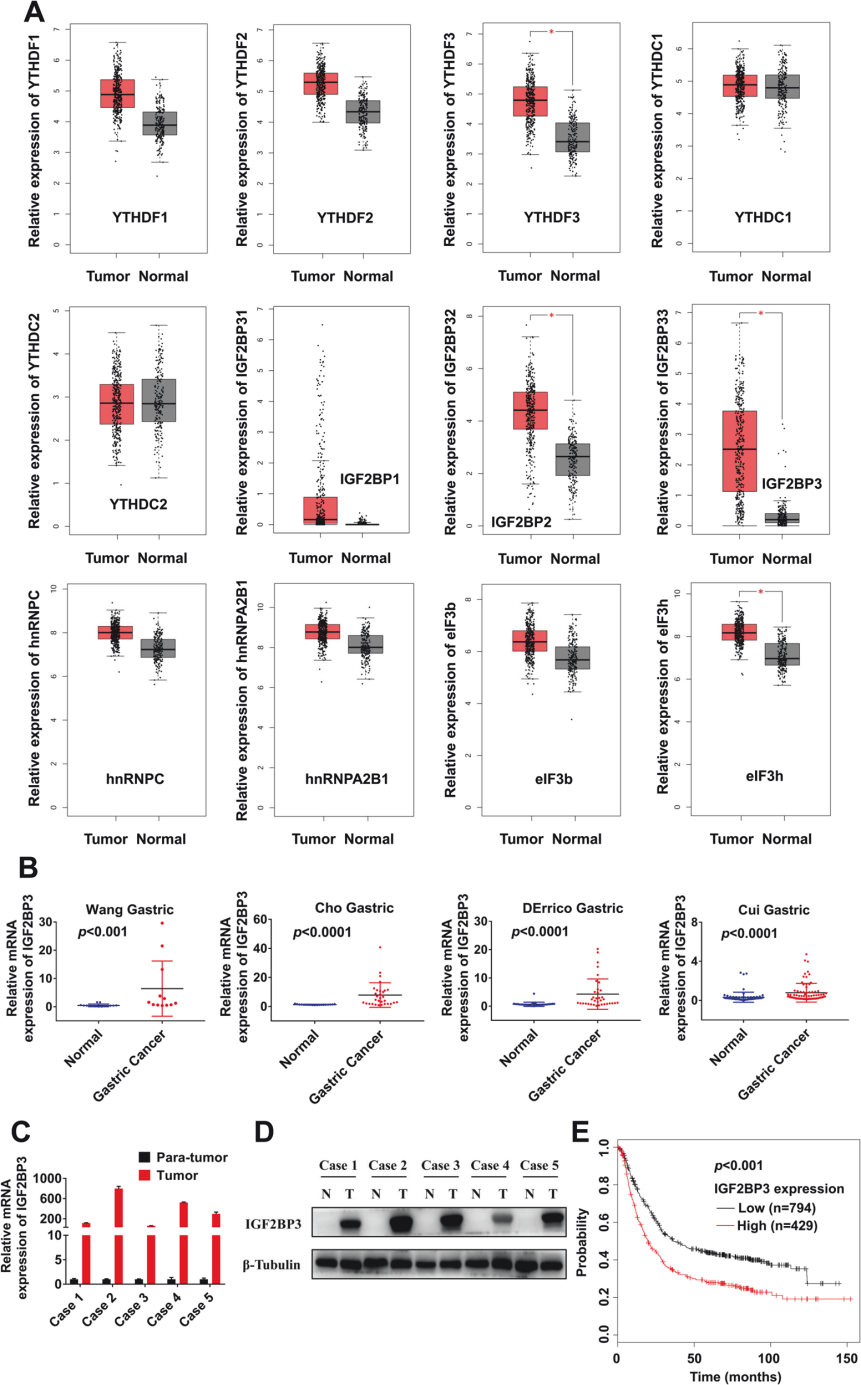
Elevated expression of m^6^A reader IGF2BP3 is associated with poor prognosis of patients with GC. **A** The mRNA expressions of YTHDF1, YTHDF2, YTHDF3, YTHDC1, YTHDC2, IGF2BP1, IGF2BP2, IGF2BP3, hnRNPC, hnRNPA2B1, eIF3b and eIF3h in GC tissues (408 cases) compared with gastric normal tissues (211 cases) in the GEPIA database. **B** The relative mRNA expressions of IGF2BP3 in GC tissues compared with gastric normal tissues in four independent datasets from the Oncomine database. **C**, **D** The relative mRNA (**C**) and protein (**D**) expressions of IGF2BP3 in tumor and para-tumor tissues of GC patients were measured by RT-qPCR and western blot analysis respectively. **E** Correlation between expression of IGF2BP3 and OS in GC patients analyzed by the Kaplan-Meier Plotter.

The m^6^A functions are depend on the recognition by m^6^A reader proteins, we further examined their expressions using the GEPIA database. Among all known m^6^A reader proteins, we found that the expressions of YTHDF3, IGF2BP2, IGF2BP3 and eIF3h in GC tissues were significantly higher than those in normal mucosa tissues respectively, particularly the differential expression of IGF2BP3 showed the most remarkable variation between GC tissues and normal mucosa tissues (Fig. 1A). Here, we also identified that the fold changes of differential expressions of IGF2BP3 were greatest between GC tissues and normal mucosa tissues, according to Wang Gastric, Cho Gastric, DErrico Gastric and Cui Gastric datasets from the Oncomine database (Fig. 1B). Five paired GC tumor and para-tumor tissues were collected to check the expressions of IGF2BP3. Consistently, RT-qPCR and western blot assays revealed that expressions of IGF2BP3 were also significantly elevated in GC tumor tissues compared to para-tumor tissues (Fig. 1C, D). We further found that GC patients with increased mRNA levels of IGF2BP3 showed poor OS using the Kaplan‐Meier Plotter (Fig. 1E). Data from the UALCAN database indicated the expressions of IGF2BP3 in GC tumor tissue with different individual cancer stage, tumor grade, nodal metastasis status and TP53 mutation status were all significantly higher than those in normal mucosa tissues respectively, and the expressions of IGF2BP3 in GC tumor tissues with TP53 mutation were upregulated compared to those without TP53 mutation (Fig. S3).

Moreover, mRNA levels of IGF2BP3 also increased in multiple different kinds of tumor (Fig. S4), indicating that IGF2BP3 may harbor as potential oncogene across multiple cancer types. The above results suggest that the m^6^A reader protein IGF2BP3 might play the pivotal role in GC progression.

### Inhibition of IGF2BP3 suppresses malignancy of GC cells

**Fig. 2:**
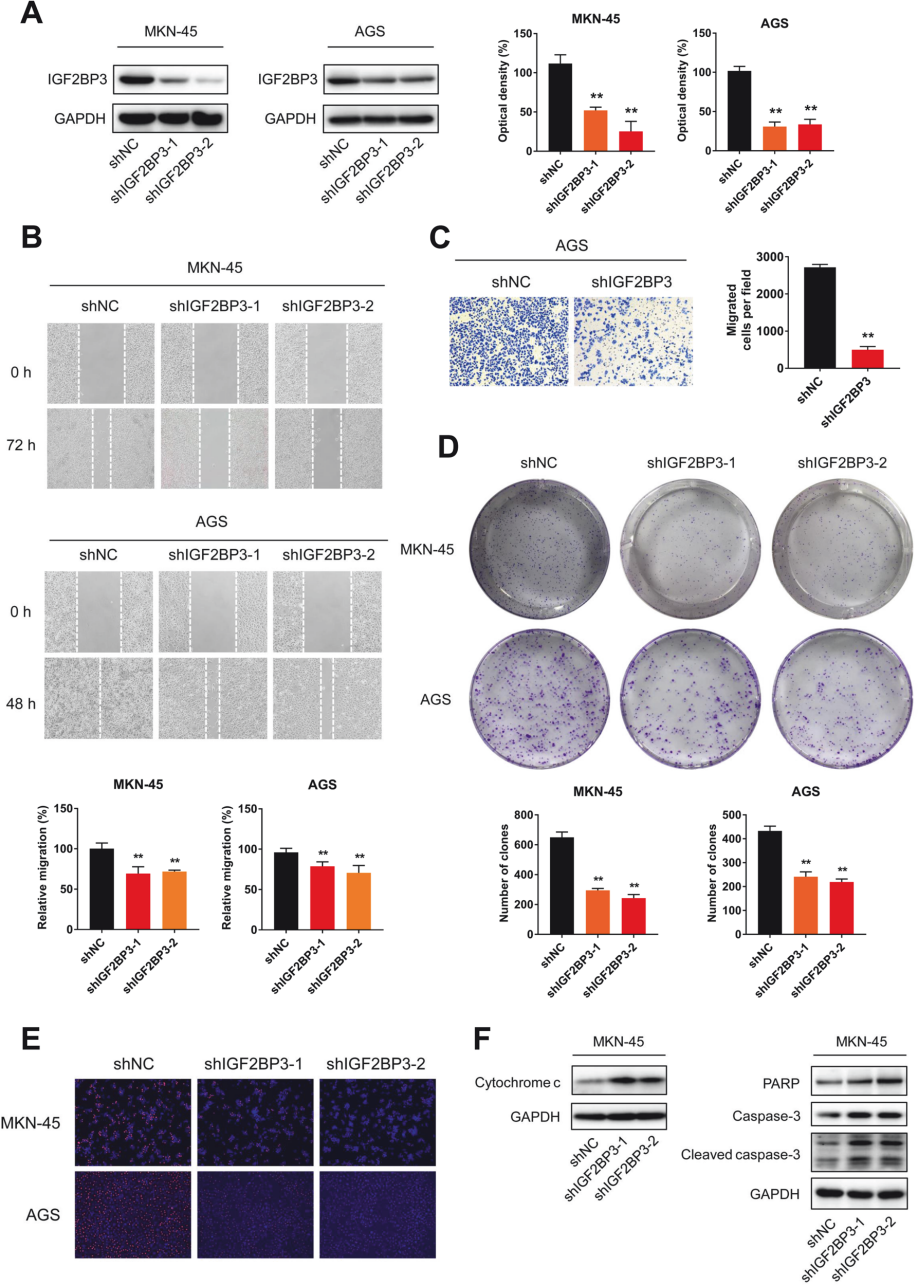
Inhibition of IGF2BP3 suppresses GC cell growth and migration in vitro. **A** The expressions of IGF2BP3 in MKN-45 and AGS cells infected with two independent shRNAs targeting IGF2BP3 or a control shRNA were measured by western blot analysis (left) and quantitatively analyzed (right), respectively. **B** Wound healing of shNC and shIGF2BP3 MKN-45 and AGS cells were recorded (up) and quantitatively analyzed (down), respectively. **C** Migration and invasion of shNC and shIGF2BP3 AGS cells were recorded (left) and quantitatively analyzed (right), respectively. **D** Colony formation of shNC and shIGF2BP3 MKN-45 and AGS cells were recorded (up) and quantitatively analyzed (down), respectively. **E** Cell proliferation of shNC and shIGF2BP3 MKN-45 and AGS cells were detected using EdU staining, respectively. **F** The expressions of Cytochrome c, PARP, Caspase-3 and Cleaved caspase-3 in shNC and shIGF2BP3 MKN-45 cells were measured by western blot analysis.

To confirm whether IGF2BP3 is an oncogenic factor in GC, we established stable IGF2BP3-knockdown GC cell lines (MKN-45 and AGS) using shRNAs (Fig. 2A). The downregulation of IGF2BP3 significantly inhibited the wound healing of GC cells (Fig. 2B) and invasion ability of AGS cells in vitro (Fig. 2C). Knockdown of IGF2BP3 obviously suppressed the colony formation and proliferation abilities of GC cells (Fig. 2D, E). In addition, knockdown of IGF2BP3 markedly provoked the expressions of apoptosis markers such as Cytochrome c, PARP, Caspase-3 and Cleaved caspase-3 in MKN-45 cells (Fig. 2F). We further inhibited the expression of IGF2BP3 by using siRNA. The results showed that knockdown of IGF2BP3 through siRNA could also significantly increase the expression of Cleaved caspase-3 in MKN-45 cells (Fig. S5A) and inhibit the wound healing of MKN-45 cells (Fig. S5B).

### Identification of potential downstream targets of IGF2BP3

**Fig. 3:**
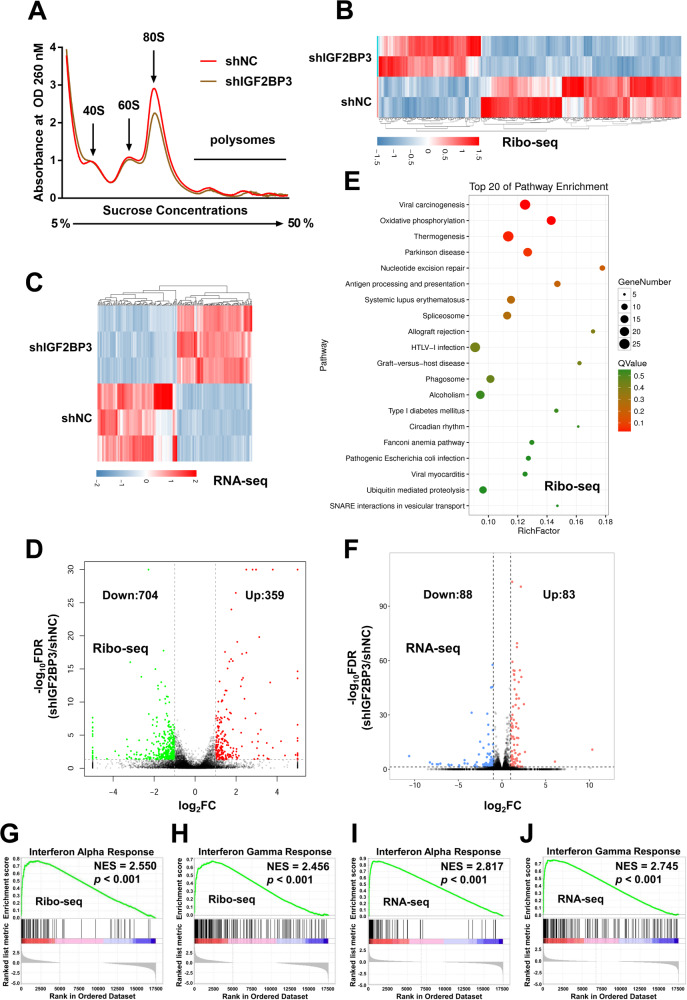
Identification of the targets of IGF2BP3 in GC cells by Ribo-seq and RNA-seq. **A** Polysome profiling of shNC and shIGF2BP3 MKN-45 cells were analyzed. **B**, **C** Differentially expressed genes (DEGs, |fold change| ≥ 2 and *p* < 0.05) between shNC (control) and shIGF2BP3 MKN-45 cells were identified by Ribo-seq (**B**) and RNA-seq (**C**) were presented in heatmaps, respectively. **D**, **E** DEGs (|fold change| ≥ 2 and *p* < 0.05) between shNC and shIGF2BP3 MKN-45 cells identified by Ribo-seq were presented in number statistics (**D**) and GO enrichment analysis (**E**). **F** DEGs (|fold change| ≥ 2 and *p* < 0.05) between shNC and shIGF2BP3 MKN-45 cells identified by RNA-seq were presented in number statistics analysis. **G**, **H** GSEA for Interferon Alpha/Gamma Response gene sets between shNC and shIGF2BP3 MKN-45 cells were identified by Ribo-seq. **I**, **J** GSEA for Interferon Alpha/Gamma Response gene sets between shNC and shIGF2BP3 MKN-45 cells were identified by RNA-seq.

IGF2BP3 as a novel m^6^A reader protein, could promote mRNA translation in a m^6^A-dependent manner. To verify whether IGF2BP3 could affect translation in GC cells, we mapped the ribosome profiles of shNC and shIGF2BP3 MKN-45 cells by separating the RNA fractions: non-translating fraction (< 40S), translation initiation fraction (＜ 80S, including 40S ribosomes, 60S ribosomes, 80S monosomes) as well as translation active polysomes (> 80S). Polysomes profiling supported the decreases of 80S monosomes assembly and polysomes in shIGF2BP3 MKN-45 cells (Fig. 3A), implying that translation of mRNAs were inhibited during IGF2BP3 knockdown. To uncover the functional downstream effectors and signaling pathways perturbed by IGF2BP3, Ribo-seq and RNA-seq were performed in shNC and shIGF2BP3 MKN-45 cells. IGF2BP3 knockdown profoundly altered the abundance of ribosome protected mRNA fragments and transcriptional gene expression (Fig. 3B, C). From Ribo-seq, we detected 359 up-regulated and 704 down-regulated transcripts in MKN-45 cells after IGF2BP3 knockdown (Fig. 3D). Gene ontology (GO) enrichment analysis of these genes unveiled several enriched pathways such as viral carcinogenesis, oxidative phosphorylation (Fig. 3E). From RNA-seq, 83 up-regulated and 88 down-regulated transcripts were identified in MKN-45 cells upon IGF2BP3 knockdown (Fig. 3F). GSEA results for 50 typical hallmark gene sets revealed that, among the up-regulated gene sets identified by Ribo-seq and RNA-seq, the IFN alpha response and IFN gamma response gene sets were all ranked in the top 2 in response to IGF2BP3 knockdown (Fig. 3G–J, Tables S5–8), indicating that IFN response related genes might be the key downstream targets of IGF2BP3 in GC. Although IGF2BP3 has been shown to play a role in promoting translation and RNA stability, considering the activation of IFN pathway could effectively inhibit GC progression and development [34–36], the up-regulated IFN response after IGF2BP3 knockdown identified by Ribo-seq and RNA-seq kindled our interest.

**Fig. 4:**
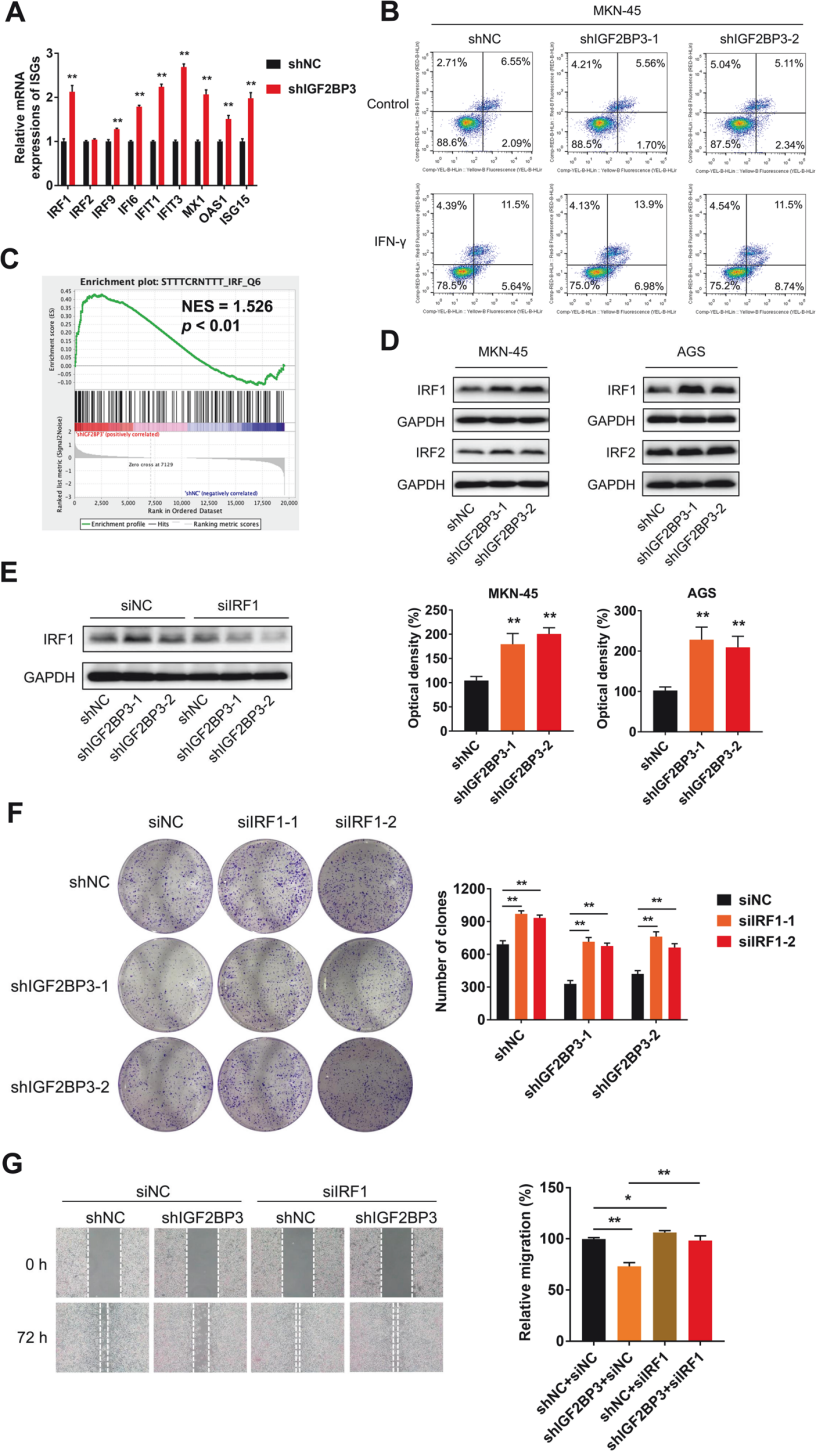
IRF1 is involved in IGF2BP3-regulated cell growth and migration in GC cells. **A** The relative mRNA expressions of IRF1, IRF2, IRF9, IFI6, IFIT1, IFIT3, MX1, OAS1 and ISG15 in shNC and shIGF2BP3 MKN-45 cells were investigated by qRT-PCR analysis. **B** Cell apoptosis of shNC and shIGF2BP3 MKN-45 cells after treated with IFN-γ (50 ng/mL) for 48 h were measured by flow cytometric analysis, respectively. **C** Gene expressions in STTTCRNTTT_IRF_Q6 set of shNC and shIGF2BP3 MKN-45 cells identified by Ribo-seq were performed with GSEA. **D** The expressions of IRF1 and IRF2 in shNC and shIGF2BP3 MKN-45 and AGS cells were measured by western blot analysis (up) and quantitatively analyzed (down), respectively. **E** The expressions of IRF1 in shNC and shIGF2BP3 MKN-45 cells after transfected with siRNA targeting IRF1 (si-IRF1_001) or a negative control siRNA (siNC) for 48 h were measured by western blot analysis, respectively. **F** Colony formation of shNC and shIGF2BP3 MKN-45 cells after transfected with siIRF1(si-IRF1_001, si-IRF1_002) or siNC for 48 h were recorded (left) and quantitatively analyzed (right), respectively. **G** Wound healing of shNC and shIGF2BP3 MKN-45 cells after transfected with siIRF1 (si-IRF1_001) or siNC for 48 h were recorded (left) and quantitatively analyzed (right), respectively.

To confirm whether IFN responses were activated after IGF2BP3 knockdown, we examined the expressions of typical interferon-stimulated genes (ISGs) by RT-qPCR analysis. We identified that expressions of several ISGs such as IRF1, IFI6, IFIT1, IFIT3, MX1, OAS1 and ISG15 were significantly up-regulated in MKN-45 cells upon IGF2BP3 knockdown (Fig. 4A), confirming that IFN responses were activated. In addition, flow cytometric analysis of sensitivity of antitumor drug revealed that knockdown of IGF2BP3 significantly promoted the apoptosis of GC cells induced by IFN-γ (Fig. 4B). These results verify that IFN pathway is the key downstream signaling regulated by IGF2BP3 in GC.

### IRF1 is involved in IGF2BP3-regulated cell growth and migration in GC cells

IRFs are the key transcription factors (TFs) involved in activating IFN responses and regulating ISGs expressions. GSEA results exhibited that, for Ribo-seq, enrichment plot of STTTCRNTTT_IRF_Q6 was significantly up-regulated in response to IGF2BP3 knockdown (Fig. 4C), displaying that IRFs might be the key downstream targets of IGF2BP3 in GC. Among all IRFs family members in mammals (IRF1 ~ IRF9), IRF1 and IRF2 play pivotal roles in carcinogenesis [15]. We detected the protein expressions of IRF1 and IRF2. Consequently, the results showed only IRF1 were commonly up-regulated in MKN-45 and AGS cells after IGF2BP3 knockdown (Fig. 4D). We further inhibited the expression of IGF2BP3 by using siRNA. As a result, we discovered that knockdown of IGF2BP3 through siRNA can significantly increase the protein expression of IRF1 in MKN-45 cells (Fig. S5A). These data suggested that IRF1 might be regulated by IGF2BP3 in GC. As a well-know tumor suppressor gene, functionally inactivating point mutation of *IRF1* was reported in GC [17], high frequency of loss of heterozygosity of the *IRF1* locus on chromosome 5q has been frequently observed in GC [16], suggesting that IRF1 plays an important role in the pathology of GC.

We next tested whether IGF2BP3 promotes GC via regulation of IRF1. Indeed, after inhibiting the expression of IRF1 by using siRNA (Fig. 4E), the effects of IGF2BP3 knockdown on GC cell wound healing and colony formation ability were abolished (Fig. 4F, G). These results suggest that IGF2BP3 promotes GC progression via regulating IRF1.

### IGF2BP3 regulates the transcription and mRNA stability of IRF1

**Fig. 5:**
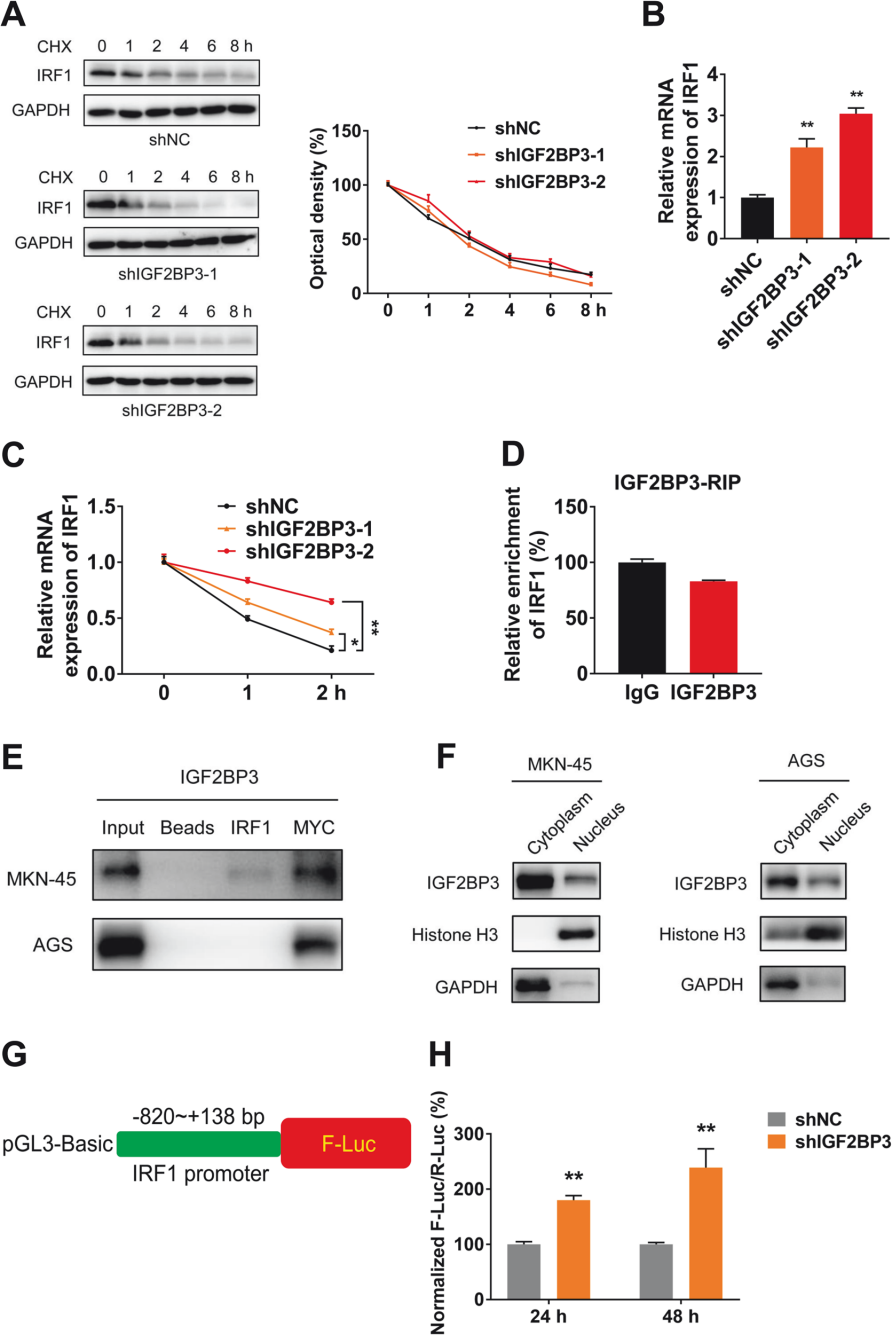
IGF2BP3 regulates the transcription and mRNA stability of IRF1. **A** The protein expressions of IRF1 in shNC and shIGF2BP3 MKN-45 cells after treated with CHX (10 μg/mL) for indicated times were examined by western blot analysis (left) and quantitatively analyzed (right). **B** The relative mRNA expressions of IRF1 in shNC and shIGF2BP3 MKN-45 cells were examined by RT-qPCR analysis. **C** The relative mRNA expressions of IRF1 in shNC and shIGF2BP3 MKN-45 cells after treated with ACTD (2 μM) for indicated times were examined by RT-qPCR analysis. **D** IGF2BP3 RIP-qPCR analysis of IRF1 mRNA in MKN-45 cells. **E** The binding capacity between IRF1 mRNA and IGF2BP3 protein in MKN-45 and AGS cells were examined by RNA pulldown and western blot analysis, respectively. MYC mRNA as the classical target of IGF2BP3 [40] was used as positive control. **F** Protein expressions of IGF2BP3 in cytoplasm and nucleus of MKN-45 and AGS cells were measured by subcellular fractionation and western blot analysis, respectively. The GAPDH and Histone H3 were used as cytoplasmic control and nuclear control respectively. **G** Schematic representation of pGL3-Basic-IRF1 promoter reporter plasmid to investigate the role of IGF2BP3 on IRF1 promoter activities. **H** shNC and shIGF2BP3 MKN-45 cells were co-transfected with pGL3-Basic-IRF1 promoter reporter plasmid and pRL-TK plasmid for 24 h or 48 h. The promoter activities were determined as a relative signal of F-luc divided by R-luc.

We examined whether IGF2BP3 regulates IRF1 expression by affecting its protein stability. MKN-45 cells after IGF2BP3 knockdown were pretreated with CHX to block protein translation. There was no obvious difference of degradation rate of IRF1 protein in the presence of CHX, suggesting that IGF2BP3-induced IRF1 expression was not related to protein stability (Fig. 5A). These results manifest that IGF2BP3 may regulate IRF1 expression in other dimension rather than at the protein level.

On the basis of the apparently increased mRNA levels of IRF1 upon IGF2BP3 knockdown (Fig. 5B), we conjectured that IGF2BP3 might modulate IRF1 at the RNA level. We treated shNC and shIGF2BP3 MKN-45 cells with ACTD to block transcription. Results showed IGF2BP3 knockdown can increase the mature mRNA stability of IRF1 in MKN-45 cells (Fig. 5C), indicating inhibition of IGF2BP3 may delay the degradation of mature mRNA of IRF1 in GC cells. However, IGF2BP3-RIP-qPCR analysis showed there was no binding capacity between IGF2BP3 protein and IRF1 mRNA in MKN-45 cells (Fig. 5D). RNA pulldown assay exhibited that there was no obvious binding ability between IRF1 mRNA and IGF2BP3 protein in GC cells (Fig. 5E). Moreover, IGF2BP3 protein and IRF1 mRNA mainly distributed in the cytoplasm and nucleus respectively by subcellular fractionation (Fig. 5F, Fig. S6). These results indicate IGF2BP3 regulates IRF1 expression instead of through directly binding with IRF1 mRNA.

To plumb whether IGF2BP3 regulates IRF1 expression through transcription control, we firstly generated the promoter-reporter of IRF1 via inserting 820 bp upstream to 138 bp downstream of transcription start site (TSS) of IRF1 to pGL3-Basic plasmid (Fig. 5G). The luciferase assay showed that the promoter activities of IRF1 in shIGF2BP3 MKN-45 cells were visibly greater than those in shNC MKN-45 cells (Fig. 5H). All these data confirm that IGF2BP3 regulates the transcription and mRNA stability of IRF1 in an indirect manner.

### NFAT1 mediates IGF2BP3-induced transcription of IRF1

**Fig. 6:**
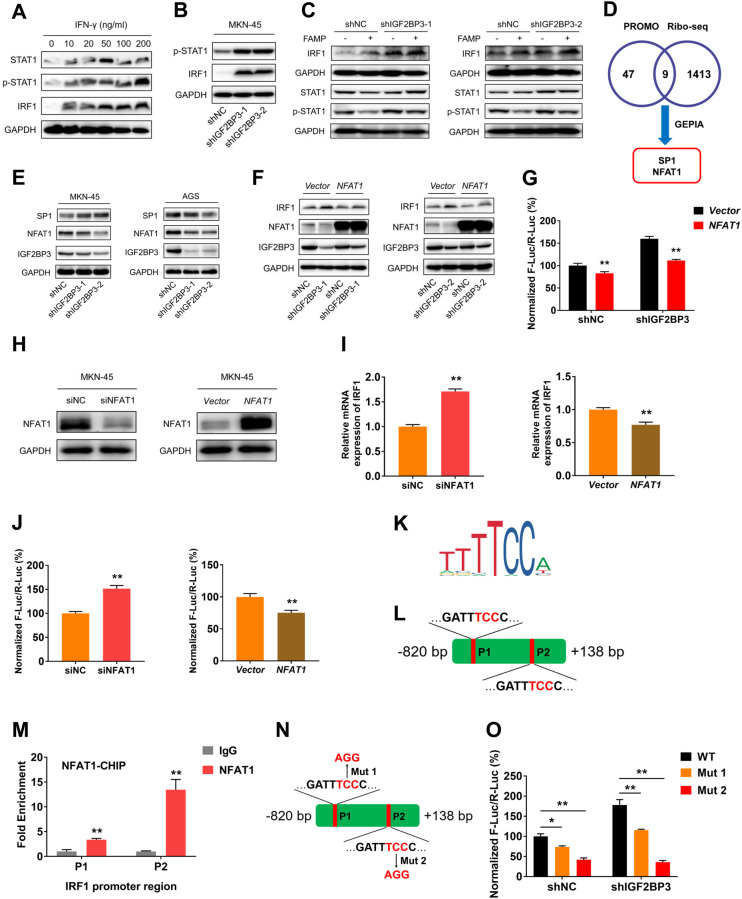
IGF2BP3 regulates IRF1 via its upstream transcription factor NFAT1. **A** The protein expressions of IRF1, p-STAT1 and STAT1 in MKN-45 cells after treated with indicated dose of IFN-γ for 48 h were examined by western blot analysis. **B** The protein expressions of IRF1 and p-STAT1 in shNC and shIGF2BP3 MKN-45 cells were examined by western blot analysis. **C** The protein expressions of IRF1, p-STAT1 and STAT1 in shNC and shIGF2BP3 MKN-45 cells after treated with FAMP for 24 h were examined by western blot analysis. **D** Overlapping genes of TFs of IRF1 predicted by the PROMO (Maximun matrix dissimilarity rate: 10%), downregulation (|fold change| ≥ 1.5) in shIGF2BP3 MKN-45 cells compared with shNC MKN-45 cells based on Ribo-seq data and upregulation in GC tissues based on the GEPIA were identified; **E** The protein expressions of SP1 and NFAT1 in shNC and shIGF2BP3 MKN-45 and AGS cells were examined by western blot analysis, respectively. **F** The expressions of IRF1 in shNC and shIGF2BP3 MKN-45 cells after transfected over expression plasmid targeting *NFAT1* or *vector* control for 48 h were measured by western blot analysis, respectively. **G** shNC and shIGF2BP3 MKN-45 cells were co-transfected with *NFAT1* over expression or *vector* control plasmid, pGL3-Basic-IRF1 promoter reporter plasmid and pRL-TK plasmid for 24 h. The promoter activities were determined as a relative signal of F-luc divided by R-luc. **H** Protein expressions of NFAT1 in MKN-45 cells were measured by western blot analysis after transfected siRNA or over expression plasmid targeting *NFAT1* for 48 h, respectively. **I** After transfected siRNA or over expression plasmid targeting *NFAT1* for 48 h, the relative mRNA expressions of IRF1 in MKN-45 cells were investigated by qRT-PCR analysis. **J** After transfected siRNA or over expression plasmid targeting *NFAT1* for 24 h, MKN-45 cells were co-transfected with pGL3-Basic-IRF1 promoter reporter plasmid and pRL-TK plasmid for 24 h. The promoter activities were determined as a relative signal of F-luc divided by R-luc. **K** The consensus sequences of TF binding sites in IRF1 promoter region were predicted by motif analysis from the JASPAR database. **L** The specific TF binding sites in IRF1 promoter region were predicted. **M** The binding between NFAT1 protein and specific TF binding sites in IRF1 promoter region in MKN-45 cells were measured by ChIP-qPCR assay. **N** Schematic representation of mutated (TCC to AGG) promoter region of pGL3-Basic-IRF1 promoter reporter plasmid to investigate the role of specific TF binding sites on IRF1 promoter activities. **O** shNC and shIGF2BP3 MKN-45 cells were co-transfected with wild-type pGL3-Basic-IRF1 promoter reporter plasmid or mutated (TCC to AGG) promoter plasmid and pRL-TK plasmid for 24 h. The promoter activities were determined as a relative signal of F-luc divided by R-luc.

We sought to unmask the molecular mechanism by which IGF2BP3 regulates transcription of IRF1. Considering the activation of STAT1 signal pathway is the major determinant for IFN-γ-induced transcription of IRF1 [37], we hypothesized that up-regulation of IRF1 by IGF2BP3 may depend on activation of STAT1. To test this, we firstly activated STAT1 by using IFN-γ. The result showed the expressions of IRF1 were concentration-dependent effects of IFN-γ (Fig. 6A). Consistent with the effects induced by IFN-γ, the expressions of IRF1 and phosphorylated STAT1 were up-regulated in MKN-45 cells upon IGF2BP3 knockdown (Fig. 6B). We treated shNC and shIGF2BP3 MKN-45 cells with Fludarabine (FAMP) to inhibit the activation of STAT1. However, no obvious attenuation of IRF1 induced by IGF2BP3 knockdown were observed (Fig. 6C). Thus, we may speculate that STAT1 was not involved in the regulation of IRF1 induced by IGF2BP3 in GC cells.

Since IGF2BP3 generally regulates gene expression in post-transcript level, we hypothesize that IGF2BP3 might regulate IRF1 by indirectly targeting its TFs. To characterize potential TFs of IRF1 involved in IGF2BP3-regulated genes, we identified two genes (SP1, NFAT1) overlapping among the predicted TFs of IRF1 by using the PROMO (http://alggen.lsi.upc.es/), downregulation of transcripts based on Ribo-seq data after IGF2BP3 knockdown and upregulation of genes in clinical GC tissues in the GEPIA database (Fig. 6D). Next, we checked the expressions of SP1 and NFAT1 in GC cells. The results showed that only NFAT1 were remarkably down-regulated both in MKN-45 and AGS cells after IGF2BP3 knockdown (Fig. 6E). These data indicated that NFAT1 might be involved in IGF2BP3-induced expression change of IRF1. Recent studies demonstrated NFAT1 significantly upregulated in GC tumor tissues compared with the adjacent non-tumor tissues, and NFAT1 signaling was activated in tumorigenesis of GC [38, 39].

To confirm the role of NFAT1 on IRF1 expression, MKN-45 cells were transfected with expression vectors of NFAT1. Our data illustrated that the overexpression of NFAT1 attenuated IGF2BP3-induced expression change of IRF1 (Fig. 6F). The dual-luciferase assay showed that overexpression of NFAT1 rescued IGF2BP3-induced change of IRF1 promoter activities (Fig. 6G). Inhibition of NFAT1 promoted the mRNA expressions and promoter activities of IRF1, and overexpression of NFAT1 decreased the mRNA expressions and promoter activities of IRF1 (Fig. 6H–J). These results suggest that NFAT1 negatively regulates the expressions and promoter activities of IRF1 in GC cells. To verify the direct combining effect of NFAT1 protein with IRF1 DNA, we identified the recognition consensus motif of NFAT1 via the JASPAR analysis (Fig. 6K). We further performed the sequence alignment in IRF1 promoter region (−820 ~ +138 bp). Our data showed there were two potential binding sites (P1, P2) of NFAT1 protein in IRF1 promoter region (Fig. 6L).

To probe the role of these binding sites in IGF2BP3-induced change of IRF1 promoter activities, we firstly examined the combining effect of NFAT1 protein with above sites in IRF1 promoter region. ChIP-qPCR assays demonstrated that NFAT1 had a remarkable enrichment of IRF1 promoter over IgG control in both P1 and P2 site (Fig. 6M), indicating a direct binding between NFAT1 and IRF1 promoter. We then testified whether above sites can regulate IGF2BP3-induced change of IRF promoter activities. We mutated the two NFAT1 potential binding sites of promoter reporter of IRF1 to generate the pGL3-IRF1-Mut 1 and pGL3-IRF1-Mut 2 (Fig. 6N). The dual-luciferase assay showed that mutation of both P1 and P2 sites attenuated IGF2BP3-induced change of IRF1 promoter activities, while the effect induced by mutation of P2 site was greater than that of P1 site (Fig. 6O). All these data suggest that NFTA1 might be responsible for the IGF2BP3-induced up-regulation of IRF1 expression in GC cells via binding to the promoter-specific sites of IRF1 to promote its transcription.

### IGF2BP3 regulates NFAT1 in a m^6^A-dependent manner

**Fig. 7:**
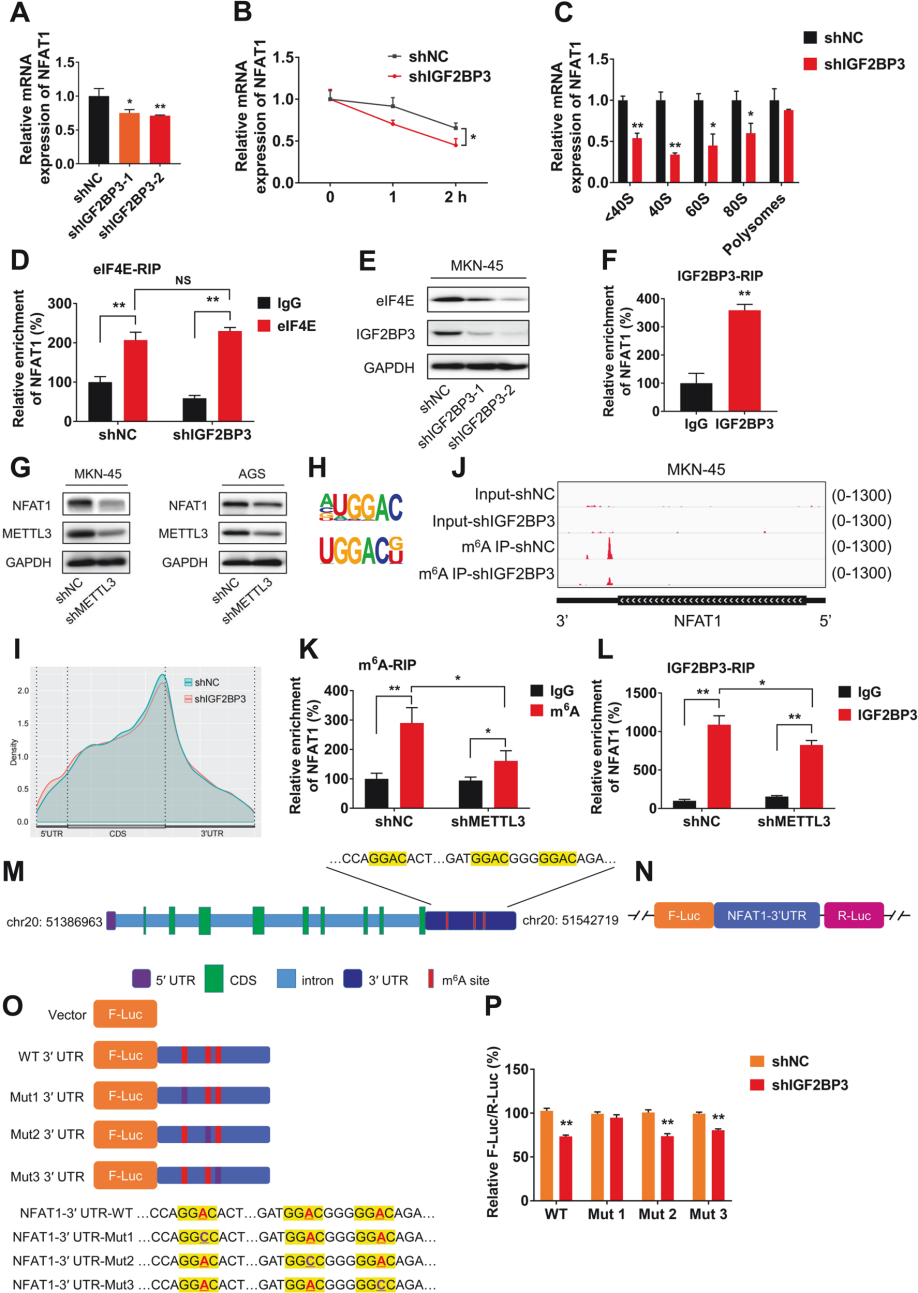
IGF2BP3 regulates NFAT1 stabilization and translation in a m^6^A-dependent manner. **A** The relative mRNA expressions of NFAT1 in shNC and shIGF2BP3 MKN-45 cells were investigated by qRT-PCR analysis. **B** The relative mRNA expressions of NFAT1 in shNC and shIGF2BP3 MKN-45 cells after treated with ACTD (2 μM) for indicated times were examined by RT-qPCR analysis. **C** The relative mRNA expressions of NFAT1 in non-ribosome portion (< 40S), 40S, 60S, 80S, and polysomes of shNC and shIGF2BP3 MKN-45 cells were examined by RT-qPCR analysis. **D** The binding between eIF4E protein and NFAT1 mRNA in shNC and shIGF2BP3 MKN-45 cells were measured by RIP-qPCR assay. **E** The expressions of eIF4E in shNC and shIGF2BP3 MKN-45 cells were measured by western blot analysis. **F** The binding between IGF2BP3 protein and NFAT1 mRNA in MKN-45 cells were measured by RIP-qPCR assay. **G** The expressions of NFAT1 in shNC and shMETTL3 MKN-45 and AGS cells were measured by western blot analysis, respectively. **H** Predominant consensus motifs GGAC were identified in shNC (up) and shIGF2BP3 (down) MKN-45 cells based on m^6^A-seq data. **I** Distribution of m^6^A peaks across mRNA transcripts in shNC and shIGF2BP3 MKN-45 cells. **J** The m^6^A signal peaks enriched in the 3’UTR of NFAT1 mRNA were identified by m^6^A-seq data. **K** The m^6^A level of NFAT1 mRNA in shNC and shMETTL3 MKN-45 cells were measured by m^6^A-RIP-qPCR assay. **L** The binding between IGF2BP3 protein and NFAT1 mRNA in shNC and shMETTL3 MKN-45 cells were measured by RIP-qPCR assay. **M** Schematic representation of potential m^6^A sites (GGAC) in NFAT1 mRNA. **N** Schematic representation of pmirGLO-NFAT1-3’UTR vector. **O** Schematic representation of mutation (GGAC to GGCC) in 3’UTR to investigate the roles of m^6^A on NFAT1 expression. **P** shNC and shIGF2BP3 MKN-45 cells were transfected with pmirGLO-NFAT1-3’UTR-WT or pmirGLO-NFAT1-3’UTR-Mut1/2/3 reporter for 48 h, the relative luciferase activities of F-Luc/R-Luc were measured.

We further investigated the potential mechanism by which IGF2BP3 regulates the expression of NFAT1. Firstly, we checked the mRNA expressions of NFAT1 in shNC and shIGF2BP3 MKN-45 cells. The results showed that mRNA levels of NFAT1 were significantly decreased in MKN-45 cells after IGF2BP3 knockdown (Fig. 7A). RNA stability assay showed IGF2BP3 knockdown decreased the mature mRNA stability of NFAT1 in MKN-45 cells (Fig. 7B), indicating inhibition of IGF2BP3 may accelerate the degradation of mature mRNA of NFAT1 in GC cells. Polysomes profiling supported that the abundance of NFAT1 mRNA in translation initiation fraction (including 40S, 60S, and 80S) of shIGF2BP3 MKN-45 cells were significantly lower than those of shNC MKN-45 cells (Fig. 7C). In addition, eIF4E-RIP-qPCR analysis showed that eIF4E had a greater enrichment capacity of NFAT1 mRNA over IgG control (Fig. 7D), indicating a direct binding between eIF4E protein and NFAT1 mRNA. Although IGF2BP3 knockdown could not affect the binding ability between eIF4E protein and NFAT1 mRNA, it significantly decreased the protein expression of eIF4E (Fig. 7E). Above data suggest that IGF2BP3 regulates the mRNA stability and translation initiation of NFAT1 in GC cells.

As a novel m^6^A reader protein, IGF2BP3 exerts its biological effects by recognizing and combining with m^6^A sites in mRNA. IGF2BP3-RIP-qPCR analysis showed that, IGF2BP3 had a greater enrichment capacity of NFAT1 mRNA over IgG control (Fig. 7F). IGF2BP3 protein and NFAT1 mRNA mainly distributed in the cytoplasm by subcellular fractionation (Fig. 5F, Fig. S6). These results indicate the direct binding between IGF2BP3 protein and NFAT1 mRNA in GC cells. To study whether IGF2BP3 regulates NFAT1 in a m^6^A-dependent manner, METTL3 knockdown stable cell lines of MKN-45 and AGS were constructed respectively by using shRNA. We found the protein expressions of NFAT1 were significantly decreased in MKN-45 and AGS cells after METTL3 knockdown, indicating that m^6^A might be relevant to IGF2BP3-induced expression change of NFAT1 (Fig. 7G). We further performed the m^6^A-seq in shNC and shIGF2BP3 MKN-45 cells, data showed GGAC motif was highly enriched within m^6^A sites in MKN-45 cells (Fig. 7H), and the m^6^A signal peaks were especially abundant in the regions of CDS and 3’ UTR (Fig. 7I). For NFAT1 mRNA, the m^6^A signal peaks were mainly enriched in 3’ UTR regions (Fig. 7J). The m^6^A-RIP qPCR assay demonstrated that NFAT1 mRNA had a remarkable enrichment capacity of m^6^A over IgG control in both shNC and shMELLT3 MKN-45 cells, and the m^6^A signal significantly weakened upon MELLT3 knockdown (Fig. 7K), indicating METTL3 could regulate the m^6^A of NFAT1 mRNA. In addition, the binding between IGF2BP3 protein and NFAT1 mRNA was remarkably decreased after METTL3 knockdown (Fig. 7L).

To explore the potential roles of m^6^A on NFAT1 expression induced by IGF2BP3, we firstly ascertained there were three potential m^6^A sites (GGAC) in NFAT1 3’UTR region (Fig. 7M). We further constructed pmir-GLO-3’UTR reporters containing wild type NFAT1 3’UTR and mutant 1/2/3 NFAT1 3’UTR (GGAC to GGCC) after the firefly luciferase reporter gene (Fig. 7N, O). The dual-luciferase assay showed that mutation of the first m^6^A site attenuated IGF2BP3-induced change of relative luciferase activity (Fig. 7P). Above results suggest that IGF2BP3 regulates the expression of NFAT1 by affecting the stability and translation of its mRNA in a m^6^A-dependent manner in GC cells.

### NFAT1/IRF1 axis is involved in IGF2BP3-regualted GC progression in vivo

**Fig. 8:**
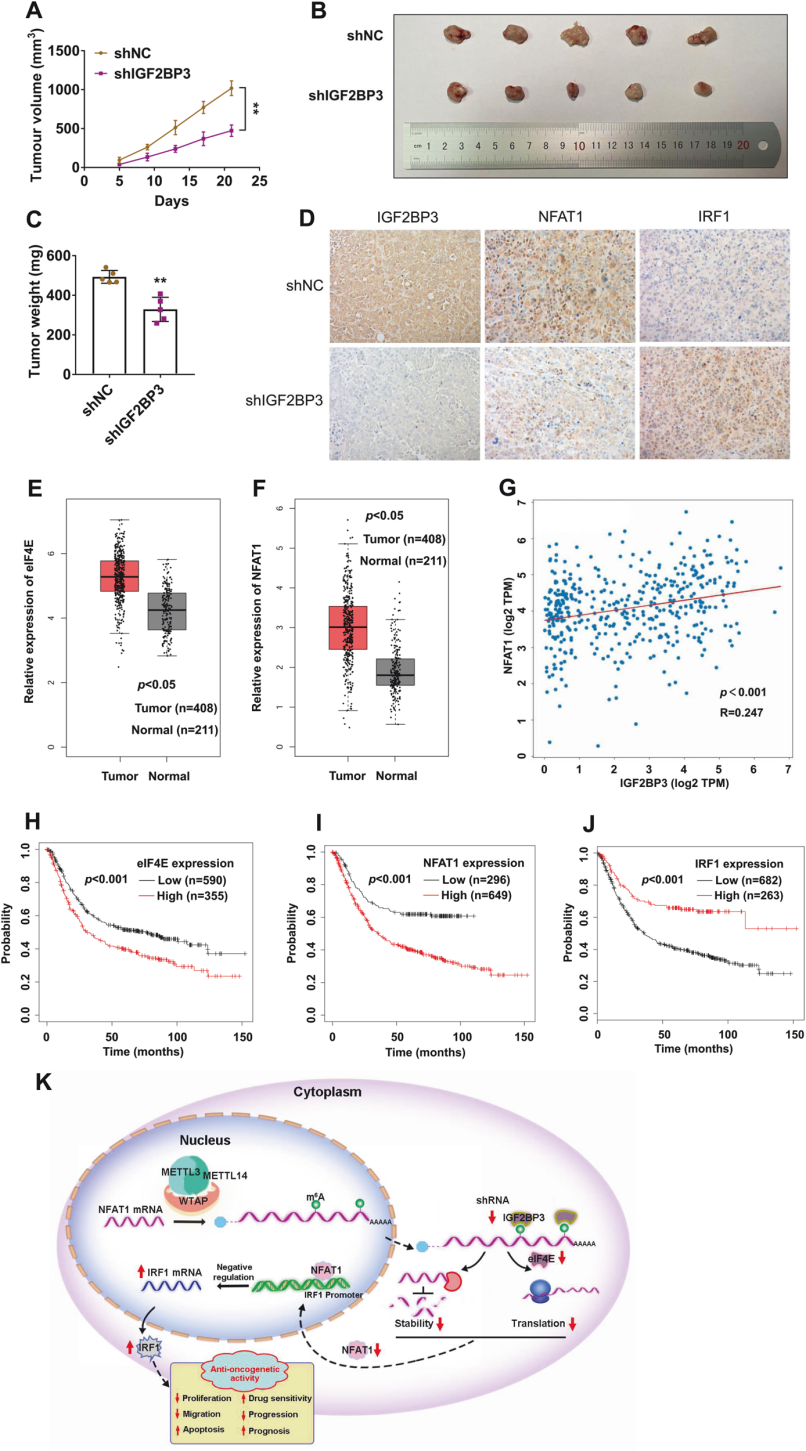
IGF2BP3/NFAT1/IRF1 axis regulate in vivo GC progression. **A** The shNC and shIGF2BP3 MKN-45 cells were subcutaneously injected into the nude mice (*n* = 5). Tumor volume was monitored every five days, and tumor growth curves were generated. **B**, **C** Images (**B**) and volumes (**C**) of xenografted tumor tissues were analyzed. **D** The expressions of IGF2BP3, NFAT1 and IRF1 in xenograft tumor tissues were examined by IHC assay. **E**, **F** The relative mRNA expressions of eIF4E (**E**) and NFAT1 (**F**) in GC tissues compared with gastric normal tissues from the GEPIA database. **G** Correlation between IGF2BP3 and NFAT1 mRNA expressions in GC tissues were analyzed from the OncoDB database. **H**–**J** Correlation between the expression of eIF4E (**H**), NFAT1 (**I**), IRF1 (**J**) and OS in GC patients were analyzed by the Kaplan-Meier Plotter. **K** The graphic illustration of IGF2BP3/NFAT1/IRF1 axis regulate GC progression.

Xenografts were used to investigate the potential effects of IGF2BP3 on GC progression in vivo. The shNC and shIGF2BP3 MKN-45 cells were injected subcutaneously into nude mice, respectively. Mice were euthanized after three weeks or when the tumor diameters were more than 2.0 cm for each group. Results showed that knockdown of IGF2BP3 markedly reduced the tumor volumes and weights of xenografts in nude mice (Fig. 8A–C), suggesting IGF2BP3 plays an oncogenic role in GC progression in vivo. IHC results showed that IGF2BP3 knockdown led to a lower level of NFAT1 and higher level of IRF1 in xenograft tumor tissues (Fig. 8D). We further found the expressions of eIF4E and NFAT1 were significantly upregulated in GC tumor tissues than normal mucosa respectively in the GEPIA database (Fig. 8E, F). In addition, there was a significantly positive correlation between the IGF2BP3 and NFAT1 mRNA expressions in tumor tissues from 414 cases with GC in the OncoDB database (Fig. 8G). Moreover, we found that the GC patients with high mRNA expressions of eIF4E and NFAT1 showed poor OS, while the GC patients with high mRNA expressions of IRF1 showed better OS by using the Kaplan‐Meier Plotter (Fig. 8H–J). Our data suggest that dysregulation of IGF2BP3-induced disorder of NFAT1/IRF1 axis might be the key factors driving GC progression (Fig. 8K).

## Discussion

Increasing studies revealed that disorder of m^6^A could trigger cancer progression [11]. As novel family of m^6^A reader proteins, IGF2BPs are composed of two RNA recognition motif (RRM) domains and four K homology (KH) domains. IGF2BP1/2/3 could regulate gene expression by promoting the mRNA stability and translation in an m^6^A-dependent manner [40]. IGF2BP3 was reported to be overexpressed in various kinds of tumor [41], indicating it might be the potential oncogene. In this study, we demonstrate that IGF2BP3 can regulate the progression of GC. In brief, IGF2BP3 was highly expressed in GC tissues and high expression of IGF2BP3 significantly associated with poor OS, indicating it might be the key factor driving GC progression. Knockdown of IGF2BP3 suppressed the in vitro migration, colony formation ability, cell proliferation and induced apoptosis of GC cells.

IGF2BP3 regulates the stability and translation of m^6^A-modified mRNA [40]. We found that knockdown of IGF2BP3 inhibited the translation of GC cells. Ribo-seq, RNA-seq and GSEA results showed that the IFN α/γ response gene sets were remarkably upregulated in response to IGF2BP3 knockdown. Particularly, IRF1 as a key TF of activating IFN responses, was up-regulated after IGF2BP3 knockdown. As a well-known tumor suppressor gene, IRF1 was reported to regulate cancer progression via suppressing proliferation and epithelial-mesenchymal transition, inducing apoptosis of cancer cells [15]. Our data showed that knockdown of IGF2BP3 suppressed the in vitro migration, colony formation ability of GC cells, which could be alleviated by the inhibition of IRF1. We identified that IRF1 was indirectly regulated by IGF2BP3, but essential in IGF2BP3-regulated GC progression.

The m^6^A reader proteins exert its functions via recognizing and binding with the m^6^A sites in mRNA. However, our data revealed that IGF2BP3 protein could not directly bind with IRF1 mRNA in GC cells. We further found knockdown of IGF2BP3 increased the mRNA expression and promoter activity of IRF1, indicating IGF2BP3 might indirectly regulate IRF1 via transcript control. Results based on Ribo-seq, TFs predictive analysis and further verification, we identified the NFAT1 was the potential TF of IRF1. Knockdown of IGF2BP3 increased the mRNA expression and promoter activity of IRF1, which could be attenuated by the overexpression of NFAT1. Results from ChIP assay verified the binding between NFAT1 and IRF1 promoter. Moreover, we identified NFAT1 prefered to bind with the specific two sites in promoter region of IRF1 and then trigger its transcript activity. Inhibition and overexpression of NFAT1 could promote and decrease the mRNA expression and promoter activities of IRF1 respectively. Our data suggest that NFTA1 negatively regulates the promoter activity and expression of IRF1 in GC cells.

To dissect the mechanism how IGF2BP3 regulate NFAT1 expression in GC, we applied the strategy by combining RIP-qPCR, polysomes profiling, m^6^A-seq and m^6^A RIP-qPCR. Results from RIP-qPCR identified the direct binding between IGF2BP3 protein and NFAT1 mRNA. Knockdown of IGF2BP3 accelerated the degradation of mature mRNA of NFAT1 in GC cells. Polysomes profiling supported that translation initiation of NFAT1 mRNA was inhibited during IGF2BP3 knockdown. Meanwhile, translation initiation factor eIF4E decreased upon IGF2BP3 knockdown. METTL3 knockdown also could down-regulate the expression of NFAT1, indicating that m^6^A might involve in IGF2BP3-induced the expression change of NFTA1. Results from m^6^A-seq and m^6^A RIP-qPCR showed that the m^6^A signal peaks of NFAT1 mRNA were enriched in its 3’ UTR region. Moreover, knockdown of METTL3 could reduce the m^6^A signal in the NFAT1 mRNA and suppress the binding between IGF2BP3 protein and NFAT1 mRNA. We further ascertained that mutation of the specific m^6^A site in NFAT1 3’UTR attenuated IGF2BP3-induced change of NFAT1 translational activities. These data support that IGF2BP3 negatively mediate IRF1 expression through regulating the stability and translation initiation of NFAT1 mRNA. Further, in vivo data suggest that IGF2BP3 plays an oncogenic role in GC progression. Clinical analysis confirms there is negative correlation between the IGF2BP3 and NFAT1 mRNA expressions in GC tissues. High expression of NFAT1 and low expression of IRF1 are associated with the reduced OS in GC patients.

Recent reports have shown that m^6^A reader protein could function as a negative regulator of IFN response to regulate innate immune response. Deletion of YTHDF2 significantly activated the type I IFN response, then increased the expression of ISGs, finally inhibited the virus replication and spread [42]. YTHDF3 negatively regulated type I IFN-mediated antiviral innate immune response, exerted its functions of promoting the virus replication; YTHDF3 defective mice effectively combated viral infections [43]. Our study provide a new insight into the function of IGF2BP3 in IFN responses, suggested IGF2BP3 may function as a negative regulator of IFN response to regulate GC progression.

In summary, our findings reveal the oncogenic role of IGF2BP3 in GC progression. Mechanistically, dysregulation of IGF2BP3-induced disorder of NFAT1/IRF1 axis is the key factor driving GC progression. IGF2BP3 regulates the stability and translation of NFAT1 mRNA in a m^6^A-dependent manner, and NFAT1 negatively mediates tumor suppressor IRF1 expression through regulating its transcription. Moreover, IGF2BP3 expression is significantly increased in GC tissues and the high expression is correlated with poor prognosis of patients with GC. Thus, IGF2BP3 could be used as a potential therapeutic target for GC. The roles of IGF2BP3 in IFN response in other types of cancer need further investigation.

### Supplementary information

Supplemental Material

Original western blots

## Data Availability

All data associated with this study are presented in the paper and its supplemental materials.
