# Supplementary material for: The RNA m6A reader IGF2BP3 regulates NFAT1/IRF1 axis-mediated anti-tumor activity in gastric cancer
Source: Cell Death Dis. 2024 Mar 6;15(3):192. doi: 10.1038/s41419-024-06566-0 (PMC10917814; doi:10.1038/s41419-024-06566-0)
Supplement: Supplementary file 1 — Supplemental Material [file 41419_2024_6566_MOESM1_ESM.pdf]

Figure S1

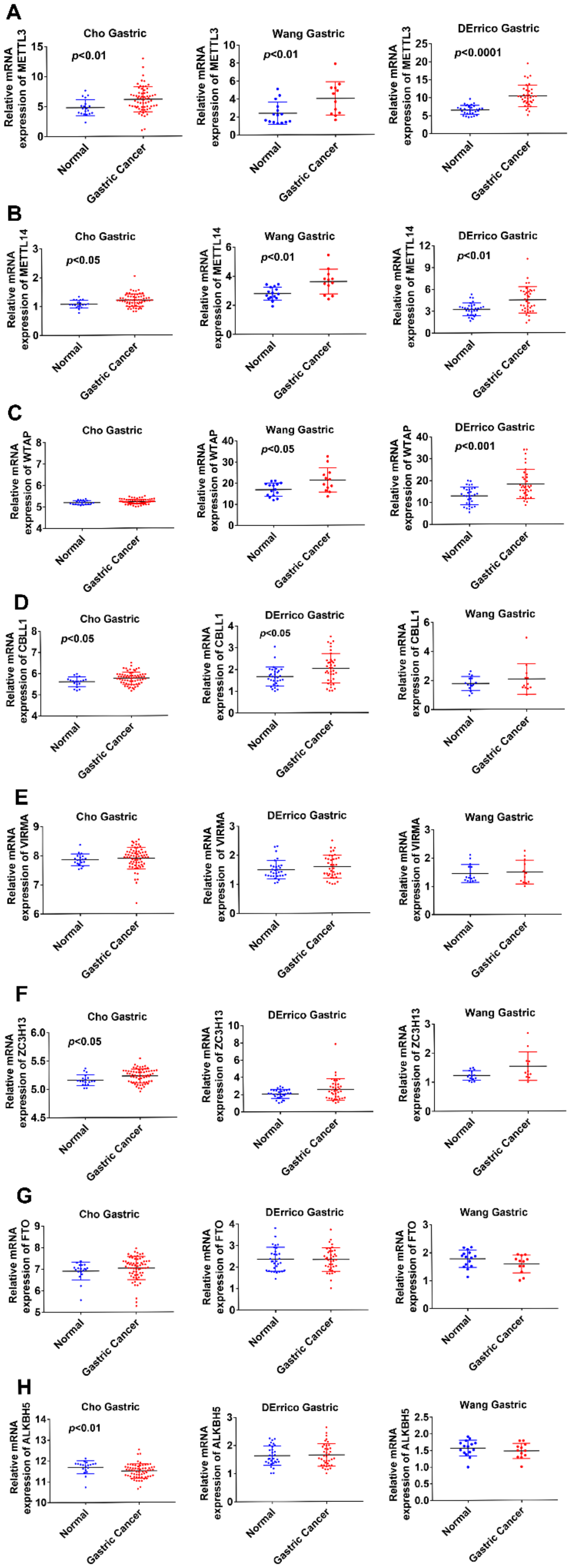
A
Cho Gastric
p<0.01
Relative mRNA expression of METTL3
Wang Gastric
p<0.01
Relative mRNA expression of METTL3
DErrico Gastric
p<0.0001
Relative mRNA expression of METTL3
B
Cho Gastric
p<0.05
Relative mRNA expression of METTL14
Wang Gastric
p<0.01
Relative mRNA expression of METTL14
DErrico Gastric
p<0.01
Relative mRNA expression of METTL14
C
Cho Gastric
Relative mRNA expression of WTAP
Wang Gastric
p<0.05
Relative mRNA expression of WTAP
DErrico Gastric
p<0.001
Relative mRNA expression of WTAP
D
Cho Gastric
p<0.05
Relative mRNA expression of CBLL1
DErrico Gastric
p<0.05
Relative mRNA expression of CBLL1
Wang Gastric
Relative mRNA expression of CBLL1
E
Cho Gastric
Relative mRNA expression of VIRMA
DErrico Gastric
Relative mRNA expression of VIRMA
Wang Gastric
Relative mRNA expression of VIRMA
F
Cho Gastric
p<0.05
Relative mRNA expression of ZC3H13
DErrico Gastric
Relative mRNA expression of ZC3H13
Wang Gastric
Relative mRNA expression of ZC3H13
G
Cho Gastric
Relative mRNA expression of FTO
DErrico Gastric
Relative mRNA expression of FTO
Wang Gastric
Relative mRNA expression of FTO
H
Cho Gastric
p<0.01
Relative mRNA expression of ALKBH5
DErrico Gastric
Relative mRNA expression of ALKBH5
Wang Gastric
Relative mRNA expression of ALKBH5
Normal
Gastric Cancer

Figure S1 Expressions of m^6A methyltransferases and demethyltransferases in GC tissues and gastric normal tissues.

(**A-H**) The mRNA expressions of METTL3 (A), METTL14 (B), WTAP (C), CBLL1 (D), VIRMA (E), ZC3H13 (F), FTO (G) and ALKBH5 (H) in GC tissues compared with gastric normal tissues in independent research projects from Oncomine database.

Figure S2

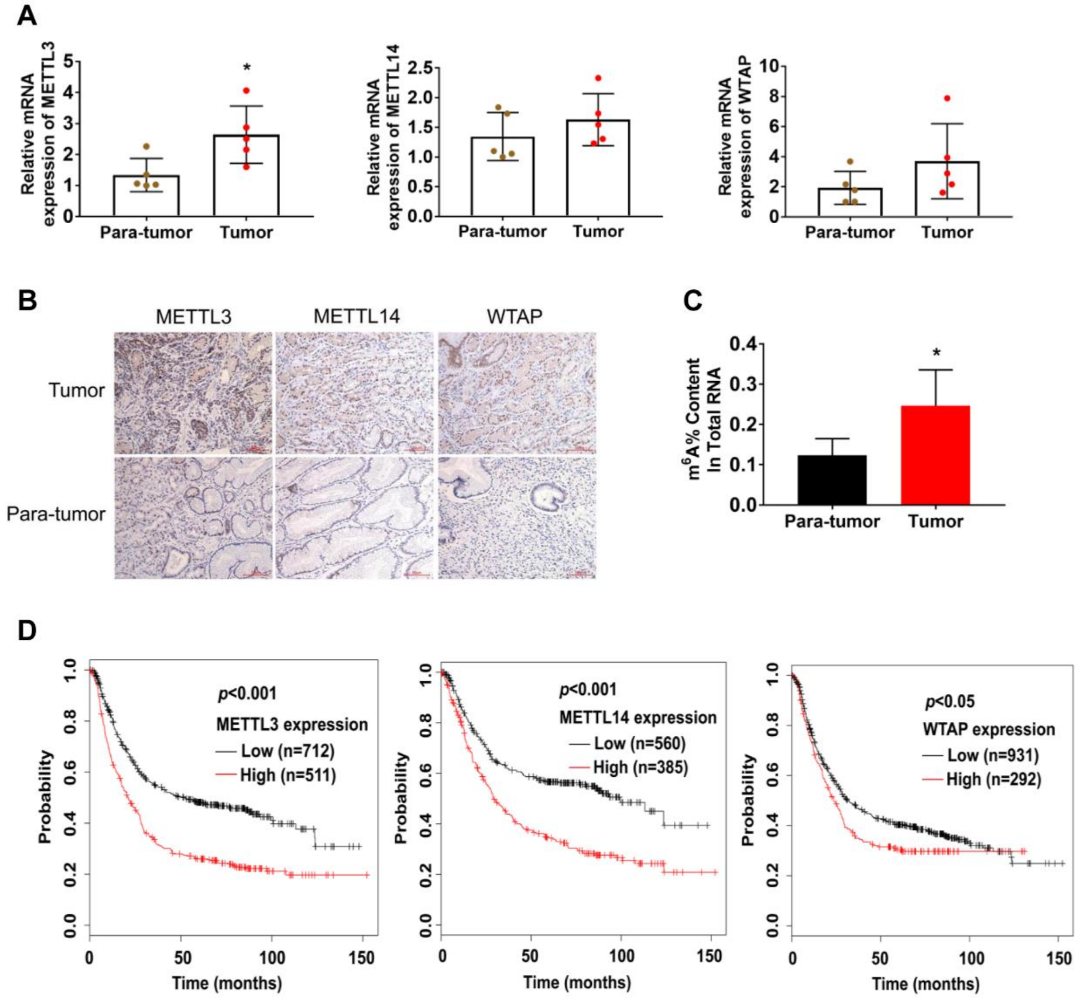

Figure S2 Expressions of m^6A methyltransferases and m^6A level in GC tissues and gastric normal tissues.

(**A**) The relative mRNA expressions of METTL3, METTL14 and WTAP in tumor and para-tumor tissues of GC patients were measured by RT-qPCR analysis respectively;

(**B**) Representative IHC (METTL4, METTL14, WTAP)-stained paraffin-embedded sections obtained from tumor and para-tumor tissues of GC patients;

(**C**) The m^{6}A levels of total RNA between tumor and para-tumor tissues (n=5) of GC patients were measured by m^{6}A Quantification Kit (Colorimetric);

(**D**) Correlation between expression of METTL3, METTL14, WTAP and OS in GC patients analyzed by Kaplan-Meier Plotter.

Figure S3

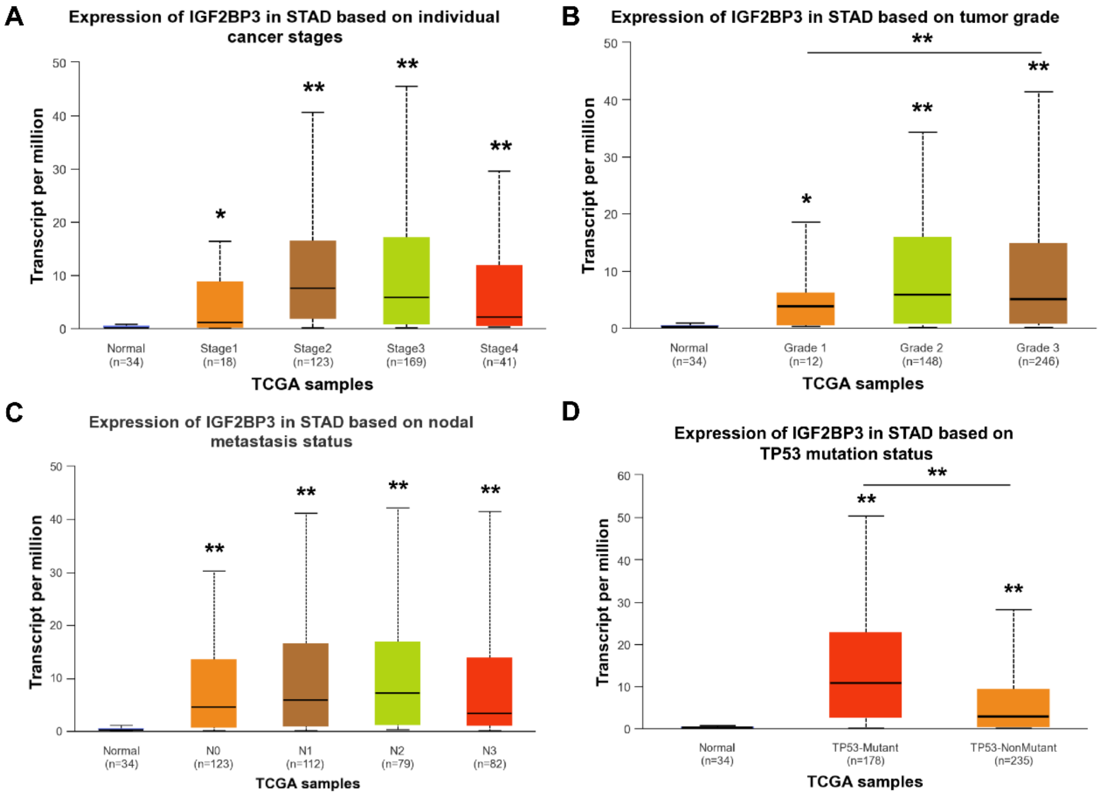

Figure S3 Clinical characteristics of IGF2BP3 in GC.

(**A-D**) The expressions of IGF2BP3 in stomach adenocarcinoma (STAD) based on

individual cancer stage (A), tumor grade (B), nodal metastasis status (C) and TP53 mutation status (D) from UALCAN database.

Figure S4

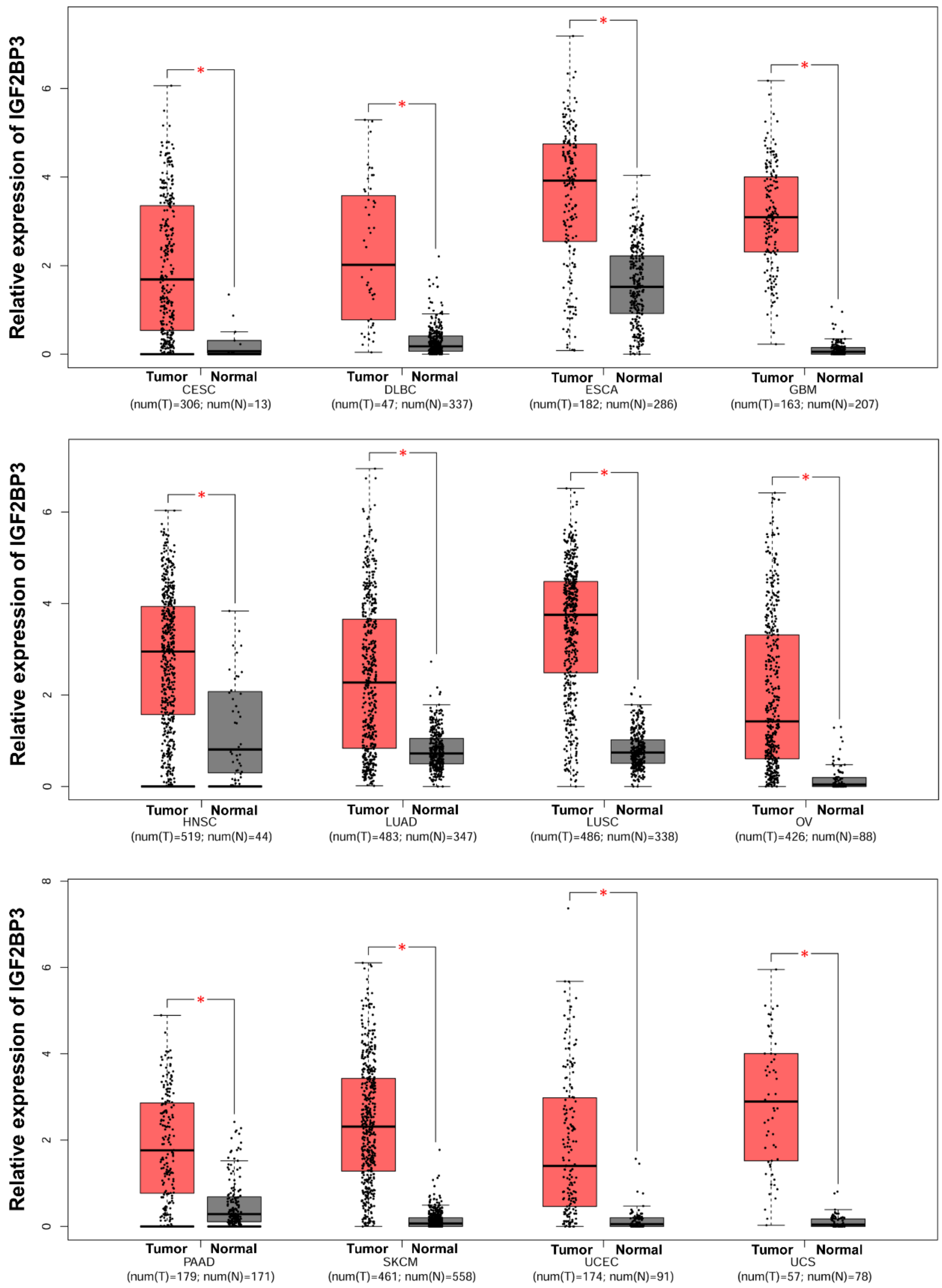

Figure S4 Expressions of IGF2BP3 in various tumor tissues and corresponding normal tissues.

The mRNA expressions of IGF2BP3 in Cervical squamous cell carcinoma and endocervical adenocarcinoma (CESC), Lymphoid Neoplasm Diffuse Large B-cell Lymphoma (DLBC), Esophageal carcinoma (ESCA), Glioblastoma multiforme (GBM), Head and Neck squamous cell carcinoma (HNSC), Lung adenocarcinoma (LUAD), Lung squamous cell carcinoma (LUSC), Ovarian serous cystadenocarcinoma (OV), Pancreatic adenocarcinoma (PAAD), Skin Cutaneous Melanoma (SKCM), Uterine Corpus Endometrial Carcinoma (UCEC) and Uterine Carcinosarcoma (UCS) tissues compared with corresponding normal tissues in GEPIA database. Red columns represent tumor tissues, gray columns represent normal tissues.

Figure S5

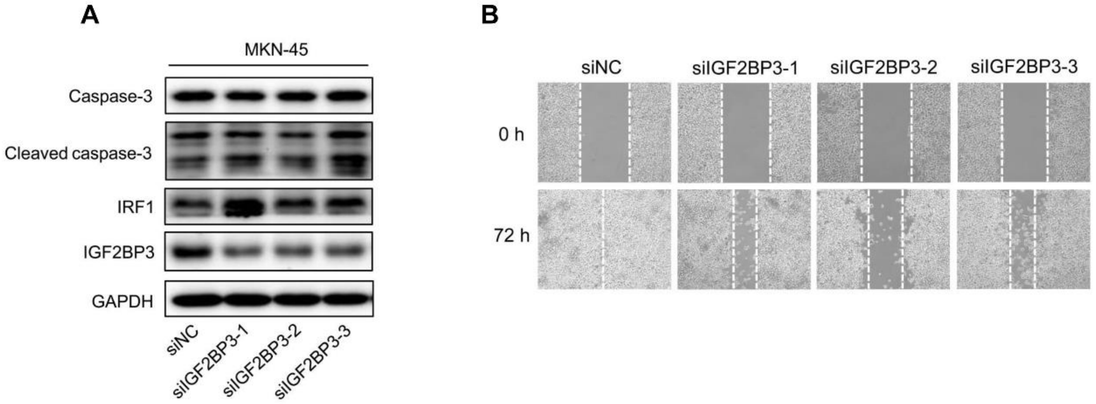

Figure S5 Effects of apoptosis and migration on MKN-45 cells induced by IGF2BP3 silencing.

(**A**) The protein expressions of IRF1, Cleaved caspase-3 and Caspase-3 in MKN-45 cells after transfected with siNC and siIGF2BP3 for 24 h were examined by western

blot analysis;

(**B)** The wound healing of MKN-45 cells after pre-transfected with siNC or siIGF2BP3 for 24 h were investigated.

Figure S6

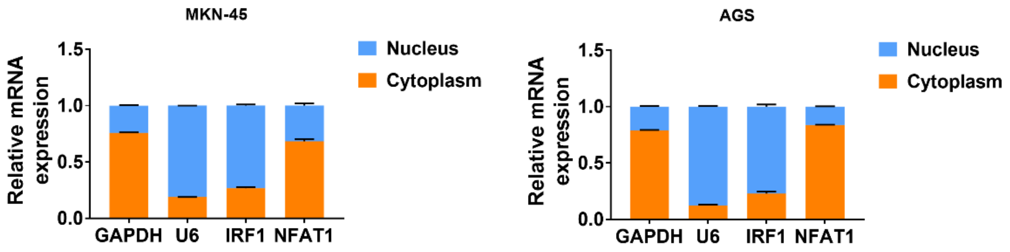

Figure S6 Expressions of IRF1 and NFAT1 in cytoplasm and nucleus of GC cells by subcellular fractionation.

The relative mRNA expressions of IRF1 and NFAT1 in cytoplasm and nucleus of MKN-45 (left) and AGS (right) cells were measured by RT-qPCR analysis respectively. The GAPDH mRNA was used as cytoplasmic control and U6 RNA as nuclear control.

Table S1. Nucleotide sequence of siRNAs and shRNAs used in this study.

Targets	Nucleotide sequence (5' - >3')
si-IGF2BP3_001	GCAGGAATTGACGCTGTATAA
si-IGF2BP3_002	CGGTGAATGAACTTCAGAATT
si-IGF2BP3_003	TGTTGTAGTCTCACAGTATAA
si-IRF1_001	GGGCTCATCTGGATTAATA
si-IRF1_002	GAAGCATGCTGCCAAGCAT
si-NFAT1	CCGAGUCCAAAGUUGUGUUUATT
sh-IGF2BP3_01	GCAGGAATTGACGCTGTATAA
sh-IGF2BP3_02	CGGTGAATGAACTTCAGAATT
sh-METTL3	CGGTGAATGAACTTCAGAATT

Table S2. Quantitative real-time PCR primers used in this study.

Targets		Nucleotide sequence (5' - >3')
Human *IGF2BP3*	Forward	GGGAGGTGCTGGATAGTTTAC
	Reverse	CTAGCTTGGTCCTTACTGGAATAG
Human *METTL3*	Forward	AAGCAGCTGGACTCTCTGCG
	Reverse	GCACTGGGCTGTCACTACGG
Human *METTL14*	Forward	GAACACAGAGCTTAAATCCCCA
	Reverse	TGTCAGCTAAACCTACATCCCTG
Human *WTAP*	Forward	TGCGACTAGCAACCAAGGAA
	Reverse	ATCTCAGTTGGGCAACGCTC
Human 18S	Forward	CGGCGACGACCCATTCGAAC
	Reverse	GAATCGAACCCTGATTCCCCGTC
Human U6	Forward	GGAACGATACAGAGAAGATTAGC
	Reverse	TGGAACGCTTCACGAATTTGCG
Human *GAPDH*	Forward	TCAACGACCACTTTGTCAAGCTCA
	Reverse	GCTGGTGGTCCAGGGGTCTTACT

Targets		Nucleotide sequence (5' - >3')
Human *IRF1*	Forward	ATGCCCATCACTCGGATGC
	Reverse	CCCTGCTTTGTATCGGCCTG
Human *IRF2*	Forward	CATGCGGCTAGACATGGGTG
	Reverse	GCTTTCCTGTATGGATTGCCC
Human *IRF9*	Forward	GCCCTACAAGGTGTATCAGTTG
	Reverse	TGCTGTCGCTTTGATGGTACT
Human *IFI6*	Forward	CAAGGTCTAGTGACGGAGCC
	Reverse	CACCCCACTGCAAGTGAAGA
Human *IFIT1*	Forward	AGAAGCAGGCAATCACAGAAAA
	Reverse	CTGAAACCGACCATAGTGGAAAT
Human *IFIT3*	Forward	AAAAGCCCAACAACCCAGAAT
	Reverse	CGTATTGGTTATCAGGACTCAGC
Human *MX1*	Forward	AGCGGGATCGTGACCAGAT
	Reverse	TGACCTTGCCTCTCCACTTATC
Human *OAS1*	Forward	AGTTGACTGGCGGCTATAAAC
	Reverse	GTGCTTGACTAGGCGGATGAG
Human *ISG15*	Forward	TGGACAAATGCGACGAACCTC
	Reverse	TCAGCCGTACCTCGTAGGTG
Human *SP1*	Forward	GTGGAGGCAACATCATTGCTG
	Reverse	GCCACTGGTACATTGGTCACAT
Human *NFAT1*	Forward	GAGCCGAATGCACATAAGGTC
	Reverse	CCAGAGAGACTAGCAAGGGG
Human *IRF1*	Forward	AAAAACAGAGGTCCTGCGTCC
Location（-711 ~ -598）	Reverse	GAAGGCAGAAAGCCTACCCTG
Human *IRF1*	Forward	CGCCGCTAGCTCTACAACA
Location（-150 ~ -33）	Reverse	CACTTAGCGGGATTCCCCA

Table S3. Primers of pGL3-Basic-IRF1 promoter-luc mutation plasmid used in this study.

Targets		Nucleotide sequence (5' - >3')
Mut 1	Forward	AGCAGCGGCCAAGTTGGGATTAGGCC<br>TGGTCCAGCAGCCCCAGA
	Reverse	TCTGGGGCTGCTGGACCAGGCCTAAT<br>CCCAACTTGGCCGCTGCT
Mut 2	Forward	CGCTAGCTCTACAACAGCCTGATTAG<br>GCCGAAATGACGGCACGCAGCCGG
	Reverse	CCGGCTGCGTGCCGTCATTTCGGCCTA<br>ATCAGGCTGTTGTAGAGCTAGCG

Table S4. Primers of pmirGLO-NFAT1-3' UTR mutation plasmid used in this study.

Targets		Nucleotide sequence (5' - >3')
Mut 1	Forward	AGCACATTCTACATGAGACAGGC<br>CACTGGAACTCTCCTGAGAAC
	Reverse	GTTCTCAGGAGAGTTCCAGTGGC<br>CTGTCTCATGTAGAATGTGCT
Mut 2	Forward	TGACTGGAGCTTGGGGGGATGGC<br>CGGGGGACAGAAGATGTGGGC
	Reverse	GCCCACATCTTCTGTCCCCCGGCC<br>ATCCCCCCAAGCTCCAGTCA
Mut 3	Forward	AGCTTGGGGGGATGGACGGGGGC<br>CAGAAGATGTGGGCACTGTGA
	Reverse	TCACAGTGCCCACATCTTCTGGCC<br>CCCGTCCATCCCCCCAAGCT

Table S5. GSEA analysis for TOP 20 up-regulated hallmarks in response to IGF2BP3 knockdown from Ribo-seq

NAME	SIZE	ES	NES	NOM *p*-val	FDR *q*-val
**HALLMARK_INTERFERON_ALPHA_RESPONSE**	93	0.77194	**2.55**	0	0
**HALLMARK_INTERFERON_GAMMA_RESPONSE**	197	0.67594	**2.456227**	0	0
HALLMARK_INFLAMMATORY_RESPONSE	197	0.473941	1.719089	0	0.00396
HALLMARK_IL6_JAK_STAT3_SIGNALING	86	0.469217	1.527786	0.004566	0.024136
HALLMARK_OXIDATIVE_PHOSPHORYLATION	183	0.416931	1.491273	0	0.030268
HALLMARK_COMPLEMENT	195	0.403726	1.468632	0	0.035152
HALLMARK_PROTEIN_SECRETION	95	0.426114	1.405172	0.009259	0.04728
HALLMARK_EPITHELIAL_MESENCHYMAL_TRANSITION	194	0.361999	1.328154	0	0.090961
HALLMARK_KRAS_SIGNALING_DN	189	0.367397	1.324348	0.028369	0.082243
HALLMARK_COAGULATION	136	0.367797	1.301858	0.015873	0.089189
HALLMARK_MTORC1_SIGNALING	196	0.347803	1.280893	0	0.096668
HALLMARK_FATTY_ACID_METABOLISM	156	0.343355	1.210959	0.046243	0.168899
HALLMARK_KRAS_SIGNALING_UP	194	0.313031	1.128164	0.103226	0.315309
HALLMARK_XENOBIOTIC_METABOLISM	199	0.304573	1.115003	0.095541	0.327271
HALLMARK_UV_RESPONSE_UP	154	0.316248	1.107088	0.160256	0.326109
HALLMARK_P53_PATHWAY	194	0.304544	1.104949	0.143885	0.310636
HALLMARK_MYC_TARGETS_V1	197	0.297477	1.095244	0.142857	0.317161
HALLMARK_APOPTOSIS	159	0.307795	1.080712	0.231707	0.342092
HALLMARK_TNFA_SIGNALING_VIA_NFKB	196	0.287681	1.047797	0.282443	0.423527
HALLMARK_BILE_ACID_METABOLISM	112	0.302873	1.030562	0.35023	0.46272

Table S6. GSEA analysis for TOP 20 up-regulated hallmarks in response to IGF2BP3 knockdown from RNA-seq

NAME	SIZE	ES	NES	NOM *p*-val	FDR *q*-val
**HALLMARK_INTERFERON_ALPHA_RESPONSE**	93	0.8610434	**2.816795**	0	0
**HALLMARK_INTERFERON_GAMMA_RESPONSE**	197	0.7545415	**2.7446244**	0	0
HALLMARK_TNFA_SIGNALING_VIA_NFKB	196	0.4738224	1.7127862	0	0.006065442
HALLMARK_IL6_JAK_STAT3_SIGNALING	86	0.50008774	1.61259	0.003039514	0.013042161
HALLMARK_INFLAMMATORY_RESPONSE	197	0.4255328	1.5330657	0	0.020052765
HALLMARK_P53_PATHWAY	194	0.373526	1.343063	0	0.09986356
HALLMARK_PROTEIN_SECRETION	95	0.3985474	1.3086156	0.05029586	0.12091216
HALLMARK_APOPTOSIS	159	0.36707816	1.2980319	0.024221454	0.116500124
HALLMARK_REACTIVE_OXIGEN_SPECIES_PATHWAY	46	0.4505283	1.2819515	0.10263158	0.11808445
HALLMARK_COMPLEMENT	195	0.34515977	1.2291671	0.06020067	0.16814537
HALLMARK_IL2_STAT5_SIGNALING	195	0.32697776	1.1684601	0.10144927	0.25343582
HALLMARK_ALLOGRAFT_REJECTION	198	0.31703052	1.1469088	0.11945392	0.27636278
HALLMARK_KRAS_SIGNALING_DN	189	0.30432373	1.1005323	0.16027875	0.36135024
HALLMARK_KRAS_SIGNALING_UP	194	0.30283365	1.0969908	0.18150684	0.34488827
HALLMARK_UV_RESPONSE_UP	154	0.28436208	0.9922355	0.4516129	0.6474104
HALLMARK_PI3K_AKT_MTOR_SIGNALING	104	0.29426554	0.98713857	0.4516129	0.6232521
HALLMARK_UNFOLDED_PROTEIN_RESPONSE	109	0.2858394	0.9594465	0.54333335	0.6872192
HALLMARK_DNA_REPAIR	140	0.26075548	0.8995156	0.7623457	0.8638948
HALLMARK_MTORC1_SIGNALING	196	0.24097143	0.8609977	0.89568347	0.92777216
HALLMARK_OXIDATIVE_PHOSPHORYLATION	183	0.23632103	0.8485225	0.9309211	0.90708554

Table S7. GSEA analysis for TOP 20 down-regulated hallmarks in response to IGF2BP3 knockdown from Ribo-seq

NAME	SIZE	ES	NES	NOM *p*-val	FDR *q*-val
HALLMARK_MITOTIC_SPINDLE	198	-0.54877603	-1.7218826	0	0.001774864
HALLMARK_G2M_CHECKPOINT	192	-0.4506889	-1.421415	0.004744959	0.11164609
HALLMARK_HEDGEHOG_SIGNALING	35	-0.56879175	-1.4140686	0.038922157	0.08444871
HALLMARK_SPERMATOGENESIS	134	-0.46700642	-1.4070473	0.009925558	0.06804475
HALLMARK_UV_RESPONSE_DN	137	-0.41864383	-1.2627765	0.056580566	0.29205137
HALLMARK_WNT_BETA_CATENIN_SIGNALING	42	-0.47184223	-1.2050877	0.1690341	0.41419125
HALLMARK_APICAL_JUNCTION	194	-0.3723395	-1.1624179	0.13706794	0.5222242
HALLMARK_MYC_TARGETS_V2	58	-0.42146432	-1.1409484	0.24324325	0.5455038
HALLMARK_NOTCH_SIGNALING	32	-0.46193683	-1.1310496	0.2760181	0.5225457
HALLMARK_E2F_TARGETS	196	-0.35517603	-1.1108228	0.22652583	0.54539543
HALLMARK_MYOGENESIS	198	-0.3388656	-1.0592307	0.33023256	0.6985335
HALLMARK_PANCREAS_BETA_CELLS	40	-0.4078308	-1.0307546	0.42419824	0.7565712
HALLMARK_HEME_METABOLISM	192	-0.3064141	-0.9519079	0.57561547	1
HALLMARK_TGF_BETA_SIGNALING	53	-0.35542414	-0.9459776	0.5557103	0.9774819
HALLMARK_APICAL_SURFACE	43	-0.3645326	-0.9398408	0.56899005	0.93474555
HALLMARK_UNFOLDED_PROTEIN_RESPONSE	109	-0.30771163	-0.90128213	0.6936488	1
HALLMARK_PEROXISOME	103	-0.29033235	-0.8524434	0.7990074	1
HALLMARK_ESTROGEN_RESPONSE_EARLY	196	-0.26752347	-0.83846563	0.88994086	1
HALLMARK_HYPOXIA	191	-0.2600811	-0.8128996	0.93286216	1
HALLMARK_ESTROGEN_RESPONSE_LATE	197	-0.2544411	-0.8008425	0.94315547	1

Table S8. GSEA analysis for TOP 20 down-regulated hallmarks in response to IGF2BP3 knockdown from RNA-seq

NAME	SIZE	ES	NES	NOM *p*-val	FDR *q*-val
HALLMARK_EPITHELIAL_MESENCHYMAL_TRANSITION	194	-0.47248012	-1.5880191	0.001356852	0.05603465
HALLMARK_MYOGENESIS	198	-0.45370814	-1.5218458	0	0.07054224
HALLMARK_APICAL_SURFACE	43	-0.5341122	-1.4245763	0.037953794	0.13794425
HALLMARK_WNT_BETA_CATENIN_SIGNALING	42	-0.48691684	-1.3225762	0.086677365	0.30329883
HALLMARK_APICAL_JUNCTION	194	-0.3990237	-1.3210044	0.022955524	0.24702702
HALLMARK_COAGULATION	136	-0.4046738	-1.301114	0.046376813	0.25055885
HALLMARK_BILE_ACID_METABOLISM	112	-0.40296268	-1.2695199	0.08018155	0.28892806
HALLMARK_UV_RESPONSE_DN	137	-0.36695355	-1.1677663	0.15988372	0.569846
HALLMARK_PEROXISOME	103	-0.37871906	-1.1666028	0.1867647	0.5102003
HALLMARK_TGF_BETA_SIGNALING	53	-0.42045006	-1.1538293	0.2272	0.50585043
HALLMARK_ESTROGEN_RESPONSE_EARLY	196	-0.3337422	-1.1111034	0.20821919	0.62177116
HALLMARK_MYC_TARGETS_V2	58	-0.3811605	-1.0769349	0.33597463	0.7121081
HALLMARK_E2F_TARGETS	196	-0.3237577	-1.0754842	0.28806585	0.6627383
HALLMARK_GLYCOLYSIS	196	-0.30165097	-1.0070286	0.42334738	0.91177493
HALLMARK_HYPOXIA	191	-0.30334234	-1.0045879	0.46638054	0.86080414
HALLMARK_ANGIOGENESIS	36	-0.37435532	-0.98192066	0.4820847	0.90297246
HALLMARK_PANCREAS_BETA_CELLS	40	-0.36515987	-0.9718265	0.4903226	0.89065015
HALLMARK_ESTROGEN_RESPONSE_LATE	197	-0.28627372	-0.9556529	0.5775249	0.9050786
HALLMARK_ADIPOGENESIS	190	-0.28414735	-0.93820137	0.60719043	0.9221536
HALLMARK_NOTCH_SIGNALING	32	-0.3688021	-0.9352291	0.55630255	0.8867994
