# Supplementary material for: The RNA m6A reader IGF2BP3 regulates NFAT1/IRF1 axis-mediated anti-tumor activity in gastric cancer
Source: Cell Death Dis. 2024 Mar 6;15(3):192. doi: 10.1038/s41419-024-06566-0 (PMC10917814; doi:10.1038/s41419-024-06566-0)

Figure 1D

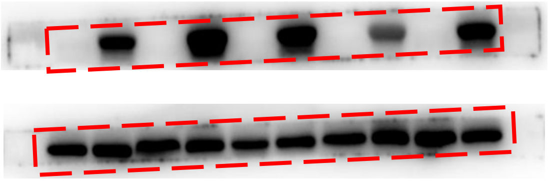

Figure 2A

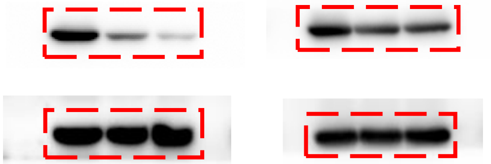

Figure 2F

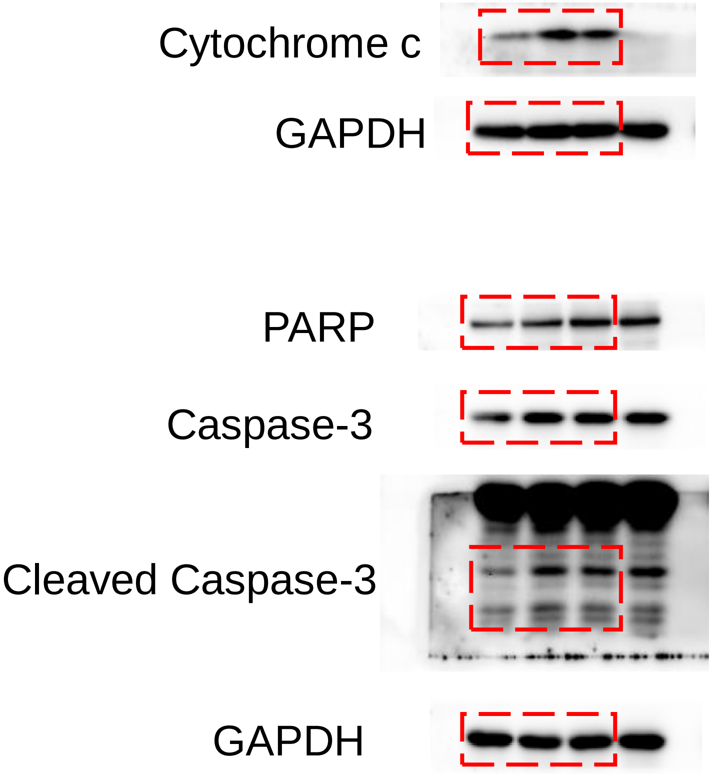

Figure 4D

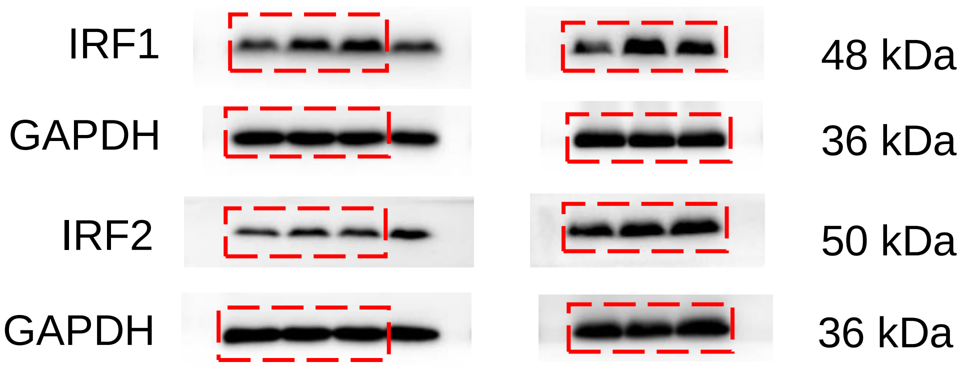

Figure 4E

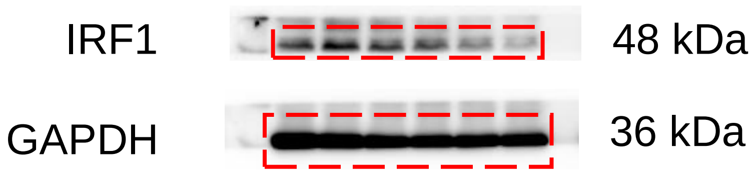

Figure 5A

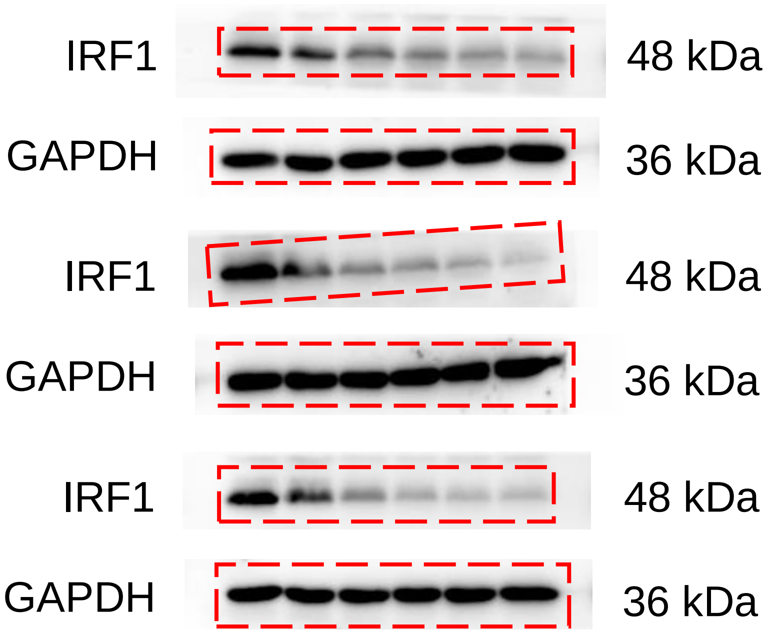

Figure 5E

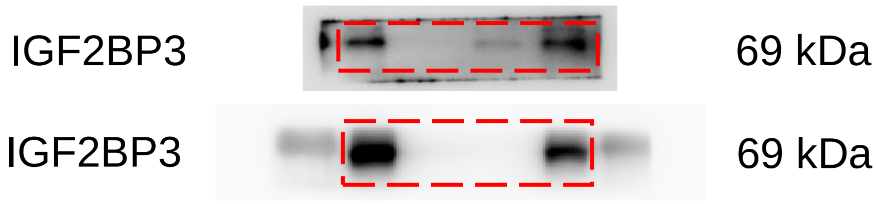

Figure 5F

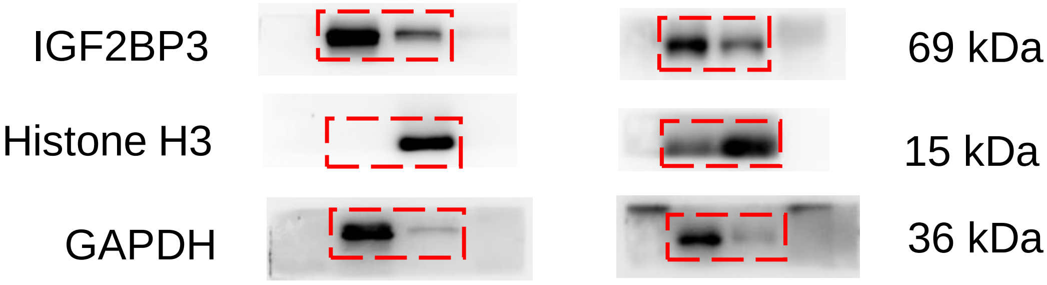

Figure 6A

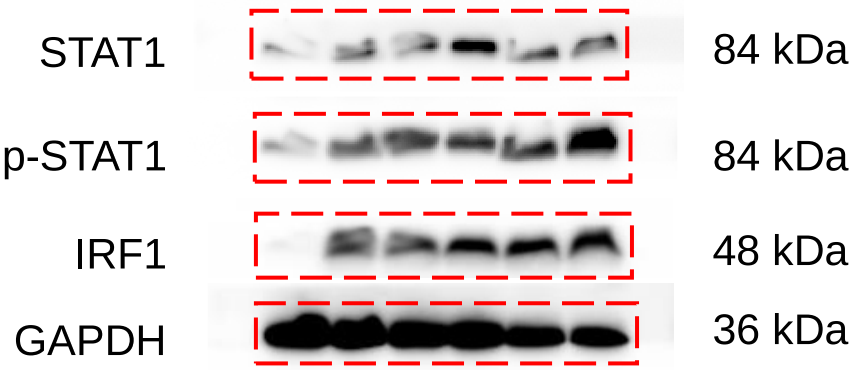

Figure 6B

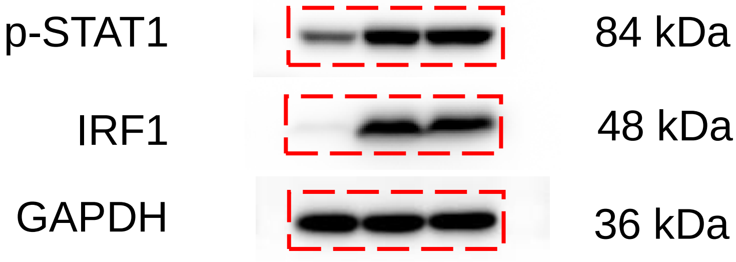

Figure 6C

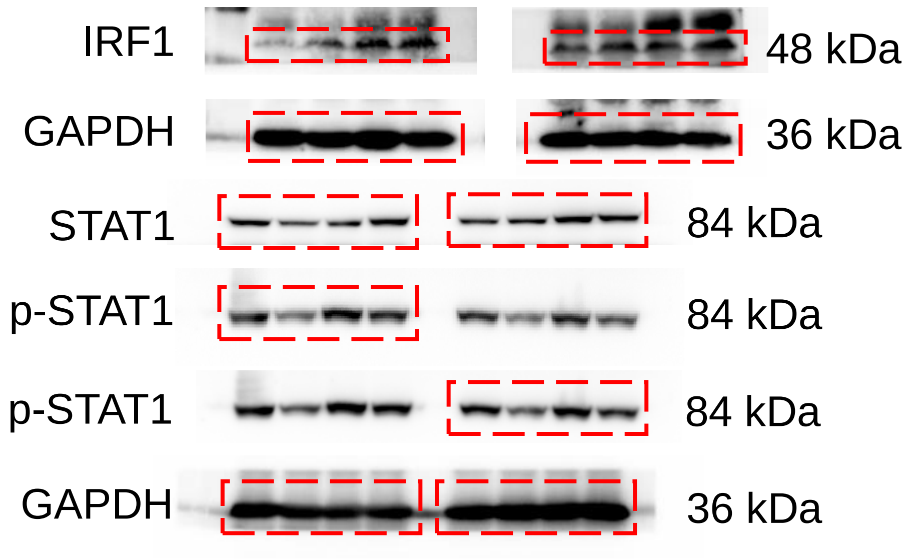

Figure 6E

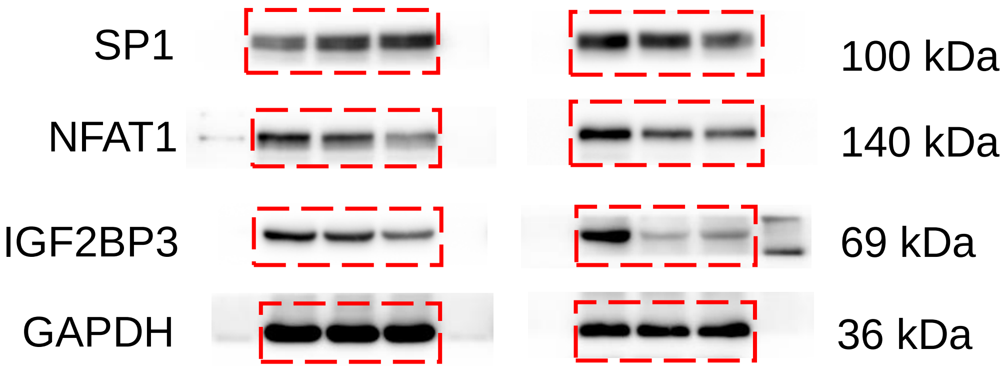

Figure 6F

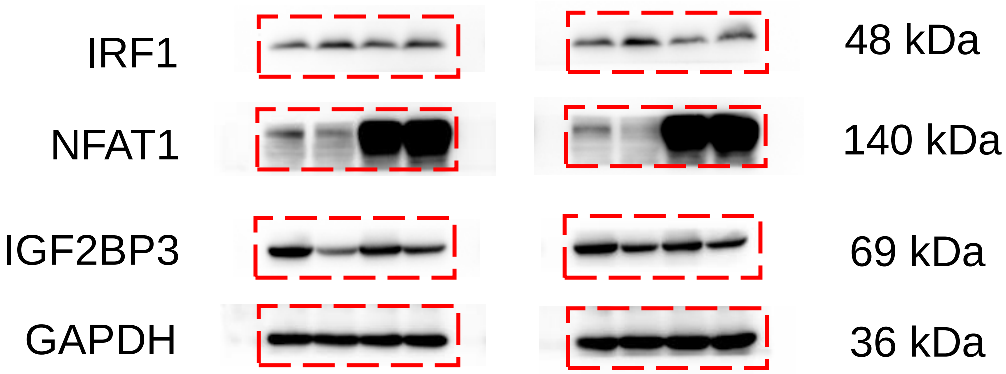

Figure 6H

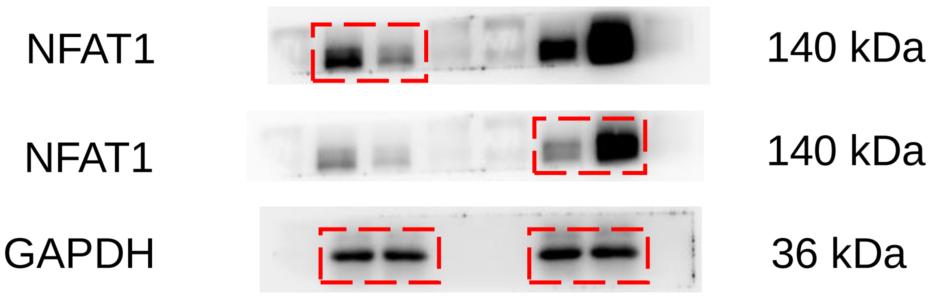

Figure 7E

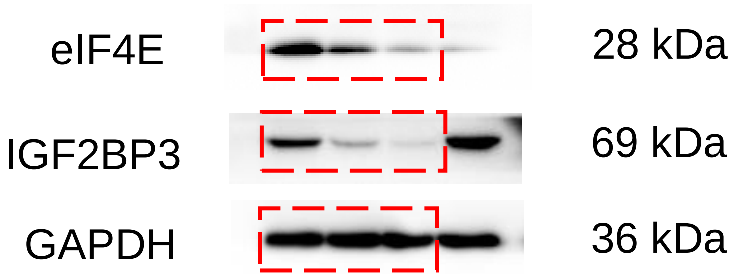

Figure 7G

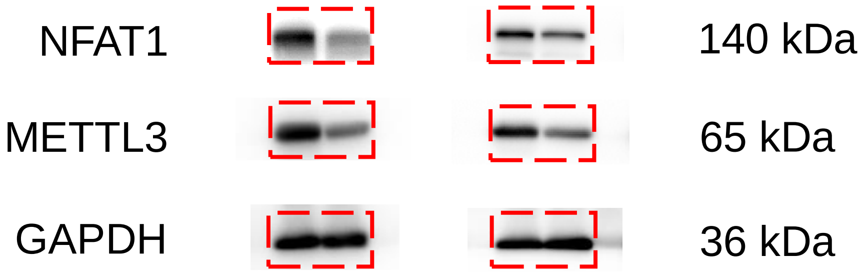

Figure S5

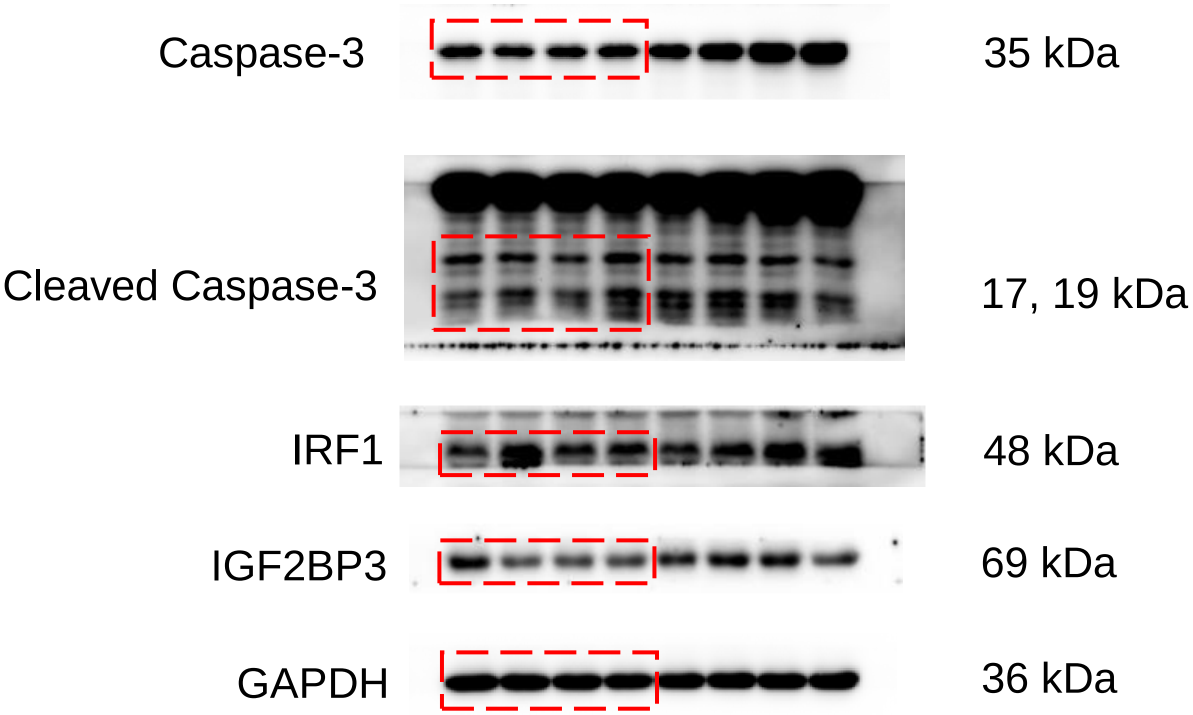

Supplement: Supplementary file 2 — Original western blots [file 41419_2024_6566_MOESM2_ESM.pdf]
